# Association between concomitant use of vancomycin-piperacillin/tazobactam and acute kidney injury in real-world setting

**DOI:** 10.3389/fphys.2026.1793497

**Published:** 2026-05-21

**Authors:** Chao-Hai Wang, Hai-Jiang Xia, Yong-Ping Fu, Nan-Nan Shen, Yue-Mei Fu

**Affiliations:** 1 Department of Pharmacy, Affiliated Hospital of Shaoxing University, Shao Xing, Zhejiang, China; 2 Department of Cardiology, Affiliated Hospital of Shaoxing University, Shao Xing, Zhejiang, China; 3 Medical Oncology, Affiliated Hospital of Shaoxing University, Shao Xing, Zhejiang, China

**Keywords:** acute kidney injury, cefepime, meropenem, piperacillin-tazobactam, vancomycin

## Abstract

**Background:**

The association between acute kidney injury (AKI) and the concomitant use of vancomycin with piperacillin-tazobactam (VPT), meropenem (VM), cefepime (VC), or monotherapy with vancomycin or piperacillin-tazobactam remains controversial. This study was conducted to compare the incidence of AKI in patients receiving VPT versus those treated with vancomycin in combination with other antibiotics or as monotherapy.

**Methods:**

A comprehensive literature search was performed across PubMed, Embase, and the Cochrane library from inception up to November 30, 2025, to identify studies reporting AKI rates among patients receiving VPT or other vancomycin-based regimens. The primary outcome was the pooled incidence of AKI, which was analyzed using a random-effects model. Subgroup analyses were carried out according to geographic region and clinical setting.

**Results:**

Thirty-five studies encompassing 39, 554 patients were included in the final synthesis. The highest pooled incidence of AKI was observed in patients receiving VPT (25.50%, 95% CI: 23.00%-28.00%), followed by VC (16.50%, 95% CI: 12.90%- 20.10%) and VM (14.00%, 95% CI: 8.90-19.20). Lower incidence rates were reported with vancomycin monotherapy (8.10%, 95% CI: 5.80%-10.40%) and piperacillin- tazobactam alone (12.30%, 95% CI: 6.70%-17.90%). Subgroup analysis showed that ICU patients experienced significantly higher AKI rates when receiving VPT (33.80%, 95% CI: 29.80%-37.70%) compared to non-ICU patients (17.20%, 95% CI: 15.80% -18.60%), a trend also observed with piperacillin- tazobactam monotherapy (*P* < 0.05). Geographically, patients in Europe exhibited a markedly higher risk of AKI with VPT (41.30%, 95% CI: 29.10%-53.50%) than those in North America (25.40%, 95% CI: 22.80-28.10) or Asia (24.80%, 95% CI: 14.60-35.00). Nevertheless, this result from European population was only based on one study, it needs to be interpreted carefully. Similarly, the risk of nephrotoxicity with vancomycin monotherapy was higher in European populations (15.70%) compared to North American (8.40%) and Asian (4.00%) patients.

**Conclusion:**

This study suggests that VPT is linked to the highest risk of AKI compared to other vancomycin-containing regimens, including beta-lactam combinations and monotherapies. These findings highlight the importance of prudent antibiotic selection and rigorous renal monitoring, particularly in critically ill patients.

## Introduction

Acute kidney injury (AKI) is a severe clinical syndrome characterized by a rapid reduction in renal function, which contributes significantly to prolonged hospitalization and increased mortality risk (Goldstein and Devarajan, 2008; Ronco et al., 2019). In hospitalized patients, the incidence of AKI can reach up to 22%, with a corresponding mortality rate of approximately 11% (Goldstein and Devarajan, 2008; Levey, 2022). Various risk factors are associated with the development of AKI, including advanced age, race, elevated baseline serum creatinine, and exposure to nephrotoxic medications (Mehta et al., 2007). Vancomycin, a widely used antibiotic for the treatment of gram-positive infections, is recognized to carry a substantial risk of nephrotoxicity (Sawada et al., 2018). The reported incidence of vancomycin-associated AKI ranges between 5% and 7% (Sawada et al., 2018). Several factors Influence AKI risk, such as dosage, serum concentrations, comorbidities, and concomitant use of other medications (Meaney et al., 2014; Kim et al., 2015). Among these factors, concurrent administration of other nephrotoxic agents has been shown to increase the likelihood of renal dysfunction (Davies et al., 2013; Contreiras et al., 2014). Evidence consistently indicates that the combination of vancomycin with other nephrotoxic drugs substantially elevates the risk of AKI (Sawada et al., 2018).

In clinical practice, vancomycin is commonly co-administered with piperacillin/tazobactam to treat severe infections, including sepsis, osteomyelitis, intra-abdominal infections, and hospital-acquired pneumonia (Jeon et al., 2017). However, evidence from observational studies suggests that the vancomycin/piperacillin -tazobactam (VPT) combination is associated with a higher incidence of AKI compared to vancomycin monotherapy or combinations with alternative antibiotics such as meropenem or cefepime (Contreiras et al., 2014; Kim et al., 2015). Consequently, clinicians prefer alternative therapeutic regimens to reduce the potential for nephrotoxicity. Recent findings further demonstrated an increased risk of AKI with VPT combination compared to vancomycin monotherapy (Giuliano et al., 2016; Hammond et al., 2017; Luther et al., 2018). These findings raise an important clinical question whether patients receiving vancomycin in combination with piperacillin/tazobactam were indeed at a higher risk of AKI compared to vancomycin monotherapy or its combination with other antibiotics, such as cefepime, or meropenem, and how to minimize nephrotoxic harm.

Despite the growing body of evidence, definitive conclusions remain difficult to establish due to the lack of randomized controlled trials (RCTs) on this topic. Current observational studies report controversial findings regarding the nephrotoxic potential of vancomycin when combined with piperacillin-tazobactam, meropenem or cefepime (Balcı et al., 2018; Mullins et al., 2018; Blevins et al., 2019). Therefore, we conducted this systematic review to compare the incidence of AKI in patients receiving VPT combination versus those treated with vancomycin monotherapy or vancomycin in combination with other beta-lactam antibiotics.

## Methods

### Data source and search strategy

A systematic literature search was conducted in three electronic databases: Pubmed, Embase, and the Cochrane library from inception to November 30, 2025, without language restrictions. The primary objective was to identify studies reporting the incidence of AKI in patients receiving VPT or vancomycin combined with other beta-lactam antibiotics such as meropenem or cefepime, or monotherapy with vancomycin or piperacillin/tazobactam. The search strategy utilized a combination of search terms, including: (“acute kidney injury” OR “AKI” OR “acute kidney failure” OR “ARF” OR “nephrotoxic”) AND (“vancomycin” OR “piperacillin/tazobactam” OR “meropenem” OR “cefepime”). Additionally, we reviewed references of included reviews to identify potential studies. The detailed search strategy is available in **Supplementary Table 1**.

### Study selection

Two investigators independently performed title and abstract of studies screening to identify potentially eligible studies. Any discrepancies were resolved through discussion and consensus, with a third author. Studies were considered eligible for inclusion if they met the following criteria: Studies reporting the occurrence of AKI in patients receiving vancomycin+ piperacillin-tazobactam, vancomycin+cefepime, vancomycin+meropenem, vancomycin, piperacillin-tazobactam; Studies design includes randomized controlled trials, cohort studies, case-control studies, or cross-sectional studies. Exclusion criteria were defined as follows: 1. Sample size fewer than 10 participants; 2. Publications lacking full-text availability, including conference abstracts, and case reports; 3. If duplicate publications derived from the same patient cohort, the most comprehensive report was retained.

### Data extraction

Data extraction was performed independently by two reviewers. Extracted data included the study title, citation details, country, study design, baseline demographic and clinical characteristics of patients, sample size, and concomitant medications, as well as other variables. All data were recorded in a pre-designed, standardized spreadsheet by two reviewers, with a confirmation by a third author. Any discrepancies were resolved through discussion by re-examining the original articles.

### Quality assessment

Methodological quality assessment was conducted by two independent investigators using a modified version of Newcastle-Ottawa Scale (NOS) to evaluate the risk of bias. These domains included representativeness of sample population, sample size, participation rate, outcome assessment, and analytical methods to control for bias (Cota et al., 2013).

Disagreements between reviewers were resolved through consensus, a third reviewer was introduced when necessary. Each item could receive a maximum score of 2 points, resulting in a total score ranging from 0 to 10 points (**Supplementary Table 2**). A score greater than 7 indicated a low risk of bias.

### Outcome of interest

The primary outcome of interest was the incidence of AKI among patients receiving combination therapy with VPT, vancomycin monotherapy, piperacillin-tazobactam monotherapy, or vancomycin in combination with ocefepime or meropenem. AKI was identified according to three established diagnostic criteria: the Acute Kidney Injury Network (AKIN) (Mehta et al., 2007), Risk, Injury, Failure, Loss of kidney function, and End-stage kidney disease (RIFLE) (Miano et al., 2022) or Kidney Disease Improving Global Outcomes (KDIGO) criteria (Khwaja, 2012).

### Statistical analysis

The overall risk of AKI was estimated by calculating pooled incidence rates with corresponding 95% confidence intervals (95% CI). A random-effects model was utilized for this study. Heterogeneity across studies was quantified using the *I^2^* statistic, with *I^2^* > 50% representing substantial heterogeneity (Tanriver-Ayder et al., 2021). Subgroup analyses were performed based on clinical setting and geographic region. Comparability within each subgroup was estimated through the interaction analyses. Sensitivity analysis was conducted by sequential exclusion of individual studies to evaluate the robustness of the pooled effect estimates. Meta-regression analysis was conducted to investigate whether baseline confounding variables significantly influenced the outcome measures. Publication bias was assessed via visual inspection of the funnel plot with Egger’ s test, when studies ≥10. For all analyses, *P* ≤ 0.05 was considered statistically significant. All the statistical analyses were performed using STATA version 13.0 (Statacorp, College Station, Texas, United States).

## Results

### Study search and selection

**Figure 1:**
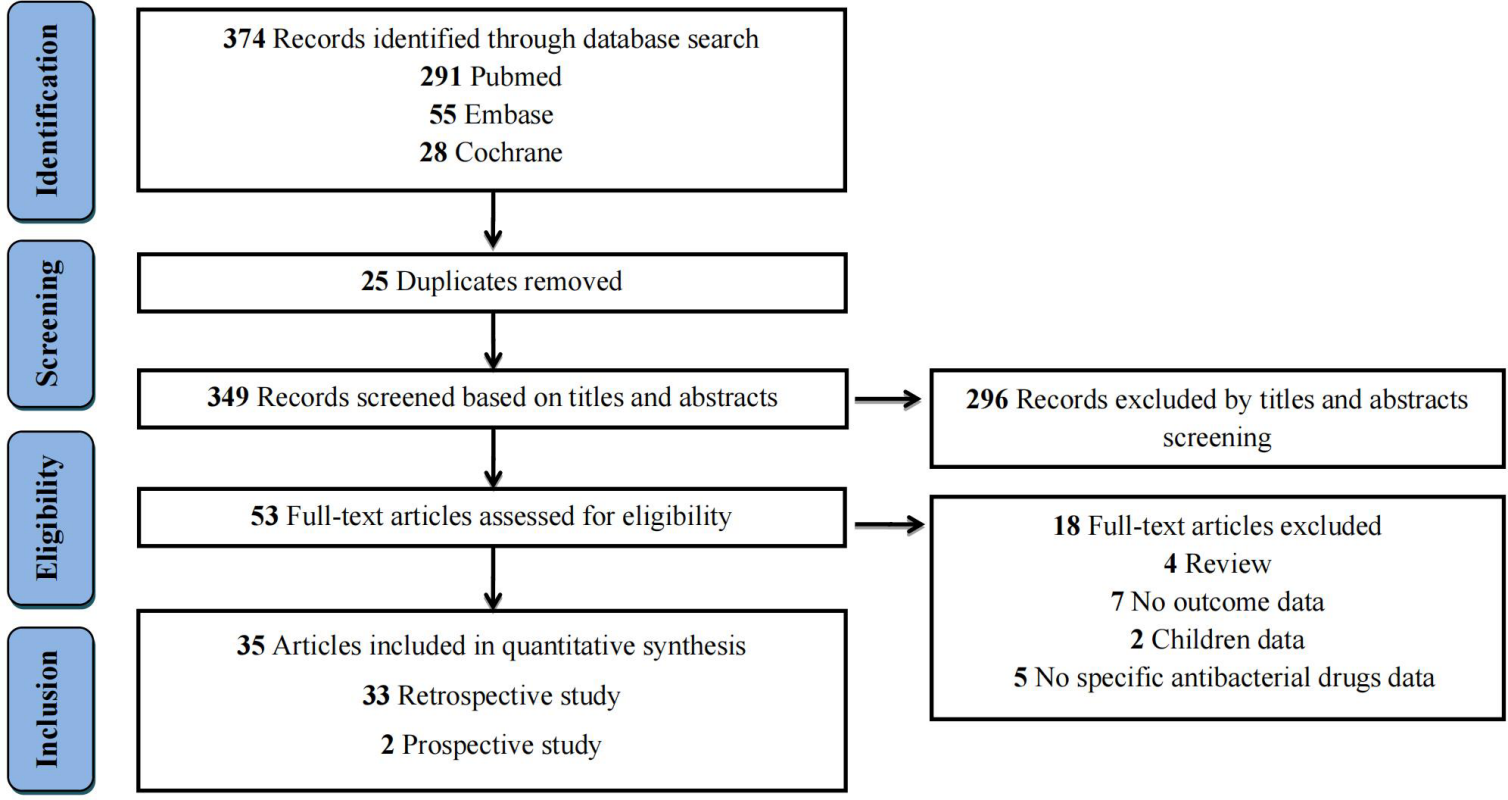
Flow diagram for the selection of eligible studies.

The literature selection process was illustrated in the flow diagram (**Figure 1**). An initial database search identified 374 records, including Pubmed (n=291), Embase (n=55), and Cochrane Library (n=28). After removing 25 duplicates, 349 records were screened for eligibility. Of these, 296 were excluded based on title and abstract evaluation. The full texts of the remaining 53 articles were retrieved and assessed in detail. Eighteen studies were further excluded for the following reasons: no specific antibacterial drugs data (n=5); absence of outcome data (n=7); review article (n=4); or children data (n=2). Detailed exclusion reasons were summarized in **Supplementary Table 3**. Ultimately, 35 articles fulfilled inclusion criteria and were included for the quantitative synthesis.

### Study characteristics

The detailed characteristics of 35 included articles were summarized in **Table 1**. Collectively, thirty-five studies encompassing 39, 554 individuals, with individual sample sizes ranging from 85 to 11, 650 patients. The majority of studies employed a retrospective design, while only two prospective investigations. Geographically, twenty-eight studies originated from North America (all conducted in the United States), six from Asia (two from China, two from Japan, one from Saudi Arabia, and one from South Korea), and one from Europe (Turkey). With regard to clinical settings, fifteen studies included both Intensive Care Unit (ICU) and non-ICU patients, nine focused on ICU populations, and eleven enrolled only non-ICU patients. The majority of studies used the AKIN criteria to identify AKI events.

**Table 1:** Detailed characteristics of the included studies.

Study	Country	Study design	AKI Definition	Clinical setting	Sample size
(Burgess and Drew, 2014)	USA	Retrospective cohort	SCr > 1.5 mg/dl or Crl < 30 ml/min	Non-ICU	191
(Gomes et al., 2014)	USA	Retrospective cohort	Kidney Disease	Non-ICU	224
(Navalkele, 2017)	USA	Retrospective cohort	Increase in creatinine by 1.5times GFR by 25%	ICU	558
(Rutter et al. 2017b)	USA	Retrospective cohort	RIFLE criteria	ICU &Non-ICU	4193
(Kim et al., 2015)	USA	Retrospective study	Increase in SCr ≥0.5 mg/dl or ≥1.5-fold	Non-ICU	228
(Meaney et al., 2014)	USA	Retrospective cohort	Increase in SCr of 0.5 mg/dl or 50% baseline	ICU &Non-ICU	125
(Rutter et al. 2017a)	USA	Retrospective study	RIFLE criteria	ICU &Non-ICU	11650
(Al Yami, 2017)	USA	Retrospective cohort	Increase in SCr by ≥0.3 mg/dl	ICU &Non-ICU	183
(Hammond et al., 2016)	USA	Retrospective cohort	Acute Kidney Injury Network	ICU	122
(Moenster et al., 2014)	USA	Retrospective cohort	Increase in SCr of 0.5 mg/dL or 50% baseline	ICU &Non-ICU	139
(Peyko et al., 2017)	USA	Prospective cohort	KDIGO acute kidney injury	ICU &Non-ICU	85
(Petite and Bauer, 2016)	USA	Retrospective cohort	KDIGO acute kidney injury	ICU &Non-ICU	417
(Anderson et al., 2017)	USA	Retrospective cohort	SCr of ≥0.3 mg/dL or ≥ 50% from baseline	Non-ICU	455
(Balci et al., 2018)	Turkey	Retrospective cohort	Glomerular filtration rate > 60 ml/min/1.73m^2^	ICU &Non-ICU	402
(Carreno et al., 2018)	USA	Retrospective cohort	Increase in SCr of 0.3 mg/L or 50%	ICU &Non-ICU	142
(Jeon et al., 2017)	USA	Retrospective cohort	Increase in Scr of ≥0.3 mg/dL or 50% baseline	ICU &Non-ICU	5335
(Cannon et al., 2017)	USA	Retrospective cohort	0.5 mg/dL increase in SCr or > 50% baseline	ICU &Non-ICU	266
(Robertson et al., 2018)	USA	Retrospective cohort	0.5 mg/dL increase in SCr or > 50% baseline	Non-ICU	169
(Mullins et al., 2018)	USA	Prospective cohort	Acute Kidney Injury Network	Non-ICU	141
(Blevins et al., 2019)	USA	Retrospective cohort	Acute Kidney Injury Network	ICU	758
(Ide et al., 2019)	Japan	Retrospective cohort	Acute Kidney Injury Network	Non-ICU	141
(Kang et al., 2019)	South Korea	Retrospective cohort	Acute Kidney Injury Network	ICU	157
(Schreier et al., 2019)	USA	Retrospective cohort	Acute Kidney Injury Network	ICU	1926
(Liu et al., 2021)	China	Retrospective cohort	Scr concentration of > 1.5-fold of baseline	ICU &Non-ICU	526
(Rungkitwattanakul et al., 2022)	USA	Retrospective cohort	Acute Kidney Injury Network	Non-ICU	207
(Tookhi et al., 2021)	Saudi Arabia	Retrospective cohort	Acute Kidney Injury Network	Non-ICU	158
(Wu et al., 2025)	China	Retrospective cohort	Acute Kidney Injury Network	ICU &Non-ICU	349
(Molina et al., 2020)	USA	Retrospective cohort	Acute Kidney Injury Network	ICU &Non-ICU	394
(Whitenack et al., 2022)	USA	Retrospective cohort	Acute Kidney Injury Network	ICU	480
(Miano et al., 2022)	USA	Retrospective cohort	Acute Kidney Injury Network	ICU	739
(Chen et al., 2023)	USA	Retrospective cohort	Acute Kidney Injury Network	ICU	3648
(Piccuirro, 2021)	USA	Retrospective cohort	Acute Kidney Injury Network	ICU	210
(Komerdelj and Buckley, 2022)	USA	Retrospective cohort	Acute Kidney Injury Network	Non-ICU	3199
(Inage et al., 2020)	Japan	Retrospective cohort	Acute Kidney Injury Network	Non-ICU	593
(Buckley et al., 2022)	USA	Retrospective cohort	Acute Kidney Injury Network	ICU &Non-ICU	1044

USA, United States Of America; AKI, acute kidney injury; GFR, glomerular filtration rate; Crl: creatinine clearance; RIFLE, end stage kidney disease; KDIGO, Improving global outcomes; ICU, Intensive Care Unit.

### Patient characteristics and quality assessment

Baseline characteristics across the included studies were summarized in **Supplementary Table 4**. The average age was 58.9 years, with 44.7% being female. Common comorbidities included hypertension (43.0%) and diabetes mellitus (29.8%). The risk bias of the included studies was summarized in **Supplementary Table 5**. All included studies demonstrated moderate to high methodological quality, with quality scores ranging from 6 to 9 score.

### AKI risk associated with VPT combination vs. monotherapy

**Figure 2:**
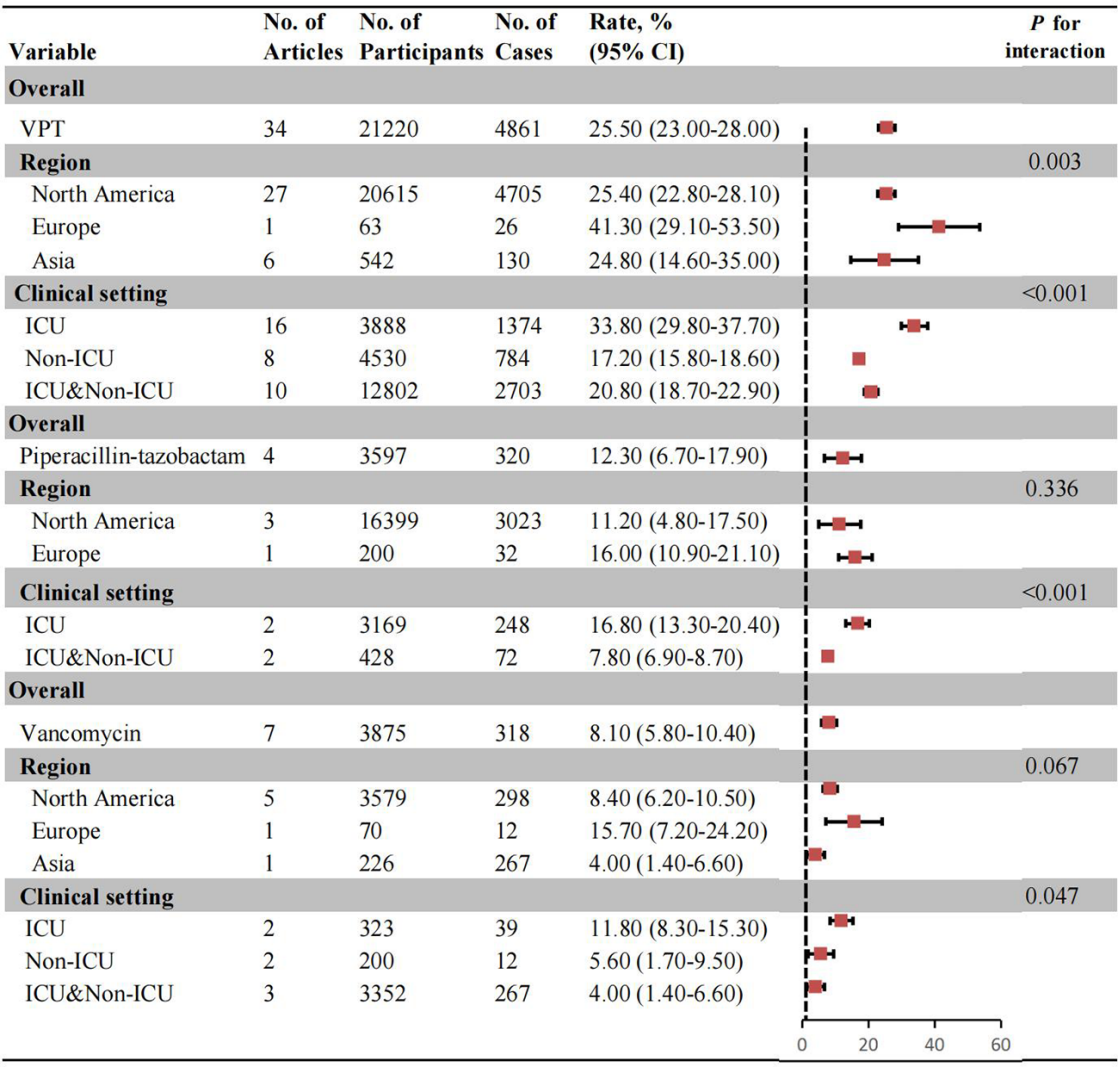
Pooled incidence of AKI in patients with Vancomycin+Piperacillin-tazobactam combination, Vancomycin monotherapy, and Piperacillin-tazobactam monotherapy. No.: number; AKI: acute kidney injury.

**Figure 3:**
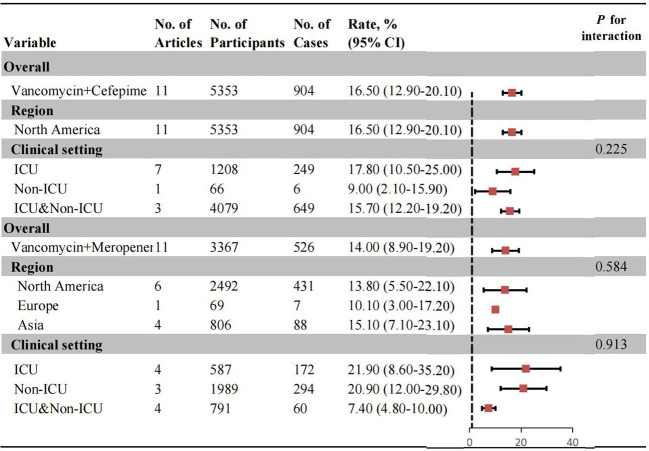
Pooled incidence of AKI in patients with Vancomycin+Cefepime, and Vancomycin+Meropenem. 95%CI, 95% conffdence interval; No., number; AKI, acute kidney injury.

The overall and subgroup AKI incidence for patients receiving VPT combination therapy versus monotherapy were summarized in **Figure 2**. The pooled analysis from thirty-four studies revealed a significantly higher AKI incidence with VPT combination therapy (25.50%, 95% CI: 23.00%-28.00%)(**Supplementary Figure 1**). Subgroup analysis by clinical settings showed that ICU patients (33.80%, 95% CI: 29.80%- 37.70%) had a higher incidence of AKI events than patients in non-ICU settings (17.20%, 95% CI: 15.80%-18.60%). In studies including both ICU and non-ICU patients, the pooled incidence of AKI was 20.80% (95% CI: 18.70%-22.90%) (**Supplementary Figure 2**). Regional subgroup analysis indicated comparable AKI rates between Northern America (25.40%, 95% CI: 22.80%-28.10%), and Asia (24.80%, 95% CI: 14.60%-35.00%) (**eSupplementary Figure 3**).

For vancomycin monotherapy, the overall AKI incidence was 8.10% (95% CI: 5.80%-10.40%) (**Supplementary Figure 4**). In subgroup analysis, ICU patients exhibited a higher AKI rate (11.80%, 95% CI: 8.30%-15.30%) than non-ICU patients (5.60%, 95% CI: 1.70%-9.50%) (**Supplementary Figure 5**). By region, no significant differences were observed across North America (8.40%, 95% CI: 6.20%-10.50%), Europe (15.70%, 95% CI: 7.20%-24.20%), and Asia (4.00%, 95% CI: 1.40%-6.60%) (**Supplementary Figure 6**). For piperacillin-tazobactam monotherapy, the overall incidence of AKI was 12.30% (95% CI: 6.70%-17.90%). Subgroup analysis revealed a higher risk of AKI in ICU patients (16.80%, 95% CI: 13.30%-20.40%) compared to ICU&Non-ICU patients (7.80%, 95% CI: 6.90%-8.70%). No significant regional differences were detected (**Supplementary Figures 7**-**9**).

### AKI risk with combination of VC and VM

The overall and subgroup AKI risk associated with vancomycin combined with cefepime (VC) or meropenem (VM) were shown in **Figure 3**. The overall AKI incidence with VC combination therapy was 16.50% (95% CI: 12.90%-20.10%). No statistically significant difference was found between ICU patients (17.80%, 95% CI: 10.50% -25.00%) and those from ICU&Non-ICU settings (15.70%, 95% CI: 12.20%-19.20%) (**Supplementary Figures 10**, **11**). For VM combination therapy, the pooled incidence was 14.00% (95% CI: 8.90%-19.20%). Subgroup analysis by clinical setting showed similar risks in ICU (21.90%, 95% CI: 8.60%-35.20%) and non-ICU patients (20.90%, 95% CI: 12.00%-29.80%). Regional analysis indicated the highest AKI incidence in Asia (15.10%, 95% CI: 7.10% -23.10%), followed by North America (13.80%, 95% CI: 5.50% -22.10%) and Europe (10.10%, 95% CI: 3.00%-17.20%) (**Supplementary Figures 12**-**14**).

### Sensitivity analysis and meta-regression

Sensitivity analyses were conducted by sequentially excluding individual studies to evaluate the influence of each study on the pooled effect estimates. The results demonstrated that no single study significantly impacted the overall incidence, thereby confirming the stability and reliability of the findings (**Supplementary Table 6**). To further investigate potential sources of heterogeneity, meta-regression analysis was performed to assess whether patient baseline characteristics have an effect on AKI incidence. No statistically significant association was observed between variables and pooled incidence (**Supplementary Table 7**).

### Publication bias

Visual inspection of funnel plots for VPT, VC, and VM combination therapies showed no apparent asymmetry, suggesting minimal risk of publication bias. This observation was corroborated by qualitative Begg’s test and Egger’s test, and yielded non-significant results, further supporting the conclusion (**Supplementary Figures 15**-**17**).

## Discussion

This study systematically evaluated the risk of AKI associated with concomitant use of vancomycin with piperacillin/tazobactam (VPT), cefepime (VC), and meropenem (VM), vancomycin or piperacillin/tazobactam monotherapy, among both critically and non-critically ill patients. Our analysis revealed that VPT significantly increased risk of AKI compared to other regimens, which aligns with findings from previous meta-analysis (Alshehri et al., 2022; Pan et al., 2025). However, previous reports have shown inconsistent results, with AKI incidence under VPT therapy ranged from 8.0% to 40% (Al Yami, 2017; Schreier et al., 2019). Furthermore, previous studies did not fully evaluate the comparative nephrotoxicity of vancomycin or piperacillin/tazobactam when used alone. Therefore, this study was conducted to provide a comprehensive assessment of AKI risk across multiple therapeutic strategies. The pooled incidence of AKI in patients receiving VPT was found to be 25.50%, rising to 33.80% in ICU settings. These findings regarding AKI risk were consistent with evidence that patients receiving VPT had a higher risk compared to vancomycin combined with alternative beta-lactams (Giuliano et al., 2016; Chen et al., 2018; Li et al., 2025). Collectively, our findings underscore that regardless of whether the comparator is monotherapy or another combination regimen, VPT remains independently associated with elevated AKI risk.

Currently, the combination of vancomycin/beta-lactam, especially piperacillin- tazobactam, is frequently prescribed for severe infections in critically ill patients (Strich et al., 2020; Aslan and Akova, 2022). However, growing concerns have emerged regarding its nephrotoxic potential, especially in ICU settings. Patients in ICU are at increased risk of AKI, and VPT-induced AKI has been linked to increased mortality (Perinel et al., 2015; Alshehri et al., 2025). Our findings are lined with previous studies (Bellomo et al., 2017; O'Callaghan et al., 2020; Tamargo et al., 2024), reinforcing the observation that the nephrotoxic effect of VPT is more pronounced in ICU compared to non-ICU settings. This risk highlights the importance of balancing antimicrobial efficacy with patient safety, particularly in ICU populations. The development of AKI in vancomycin-treated patients is multifactorial, influenced by critical illness severity, sepsis, hemodynamic instability, exposure to contrast agents, and concurrent administration of other nephrotoxic drugs (Alaradi and Albariqi, 2025; Chen et al., 2025). Importantly, reducing both administration and duration of vancomycin and piperacillin-tazobactam could help lower the likelihood of AKI. Therefore, based on current real-world evidence, clinicians should carefully consider the AKI risk caused by VPT when formulating antimicrobial regimens in ICU patients.

The identification of risk factors for AKI is crucial in clinical practice, yet challenges remain due to the lack of accurate diagnostic tools (Chen et al., 2021). Existing literature suggests that advanced age, history of kidney diseases, and concomitant use of nephrotoxic medications, such as non-steroidal anti-inflammatory drugs, which are associated with higher AKI susceptibility (Yousif et al., 2023). A large pharmacoepidemiologic study emphasized that in ICU patients, the presence of multiple concurrently administered nephrotoxic drugs introduces significant confounding, making it difficult to isolate the renal impact of individual agents (Yasrebi-de Kom et al., 2023). Therefore, various clinical measures have been adopted to mitigate AKI risk during vancomycin therapy. These include avoiding unnecessary co-administration of nephrotoxic drugs and implementing therapeutic drug monitoring for vancomycin to optimize dosing and maintain trough levels within a safe range (Watkins and Deresinski, 2017). The specific clinical strategies to mitigate the risk of acute kidney injury (AKI) include maintaining optimal fluid balance, selecting alternative antibiotics for patients at elevated risk, and minimizing the duration of combination antimicrobial therapy.

AKI is characterized by aggressive inflammation and renal cell destruction, leads to abrupt kidney impairment (Bagga et al., 2007; Fang et al., 2010). Piperacillin/tazobactam alone has been associated with relatively low nephrotoxicity, its combination with vancomycin markedly increases renal risk (Le Moyec et al., 2002; Takada et al., 2025). Despite epidemiologic data indicating increased nephrotoxicity with VPT, the precise biological mechanisms remain incompletely understood, with only hypothetical pathways proposed. Evidence from animal and human studies highlights cystatin-C as a promising biomarker for early detection of potential nephroprotection (Pais et al., 2020; Chang and Pais, 2022; Miano et al., 2022). Vancomycin-induced nephrotoxicity is primarily attributed to oxidative stress within renal tubular cells, whereas VPT combination therapy is frequently associated with acute interstitial nephritis (McKamy et al., 2011; Pratt et al., 2014). Another plausible mechanism involves reduced renal clearance of vancomycin when co-administered with piperacillin/tazobactam, leading to drug accumulation (Burgess and Drew, 2014; Gomes et al., 2014). Additionally, VPT use has been shown to elevate urinary levels of kidney injury biomarkers TIMP2 and IGFBP7, indicating the early sign of renal tubular toxicity (Kane-Gill et al., 2019). VPT may inhibit proximal tubule creatinine secretion, its competition there could explain some low-stage AKI (Schreier et al., 2019). Additionally, piperacillin exacerbates vancomycin-induced nephrotoxicity (Takada et al., 2025). Vancomycin triggers apoptosis through caspase-3/7 activation, but piperacillin alone does not activate this pathway but synergistically reduces cell viability when co-administered with vancomycin. Furthermore, piperacillin amplifies vancomycin-induced upregulation and secretion of neutrophil gelatinase-associated lipocalin (NGAL), a well-validated urinary biomarker of AKI, thereby providing functional evidence of complementary cytotoxic effects on renal proximal tubular cells (Malhotra and Siew, 2017). It is essential to evaluate the risk and benefit of vancomycin with piperacillin/tazobactam. Two recent studies have explored whether there is a clinical net benefit in VPT combination therapy, and the result was valuable (Giuliano et al., 2016; Hammond et al., 2017). In line with these insights, our study confirms that VPT is associated with a significantly elevated risk of AKI.

Several limitations should be acknowledged in the present study. First, most of included studies were retrospective in design, which inevitably carries a risk of selection and publication bias. Sensitivity analyses were performed by sequentially excluding individual studies, it could partly mitigate potential bias. Second, variations in comorbid conditions and concomitant medication use, including non-steroidal anti-inflammatory drugs, proton-pump-inhibitors, and diuretics, may act as unmeasured con-founders, potentially influencing the observed association. Third, heterogeneity in outcome definitions across studies may affect the consistency of pooled estimates, however, we applied random-effect model to minimize this impact. Fourth, the criteria for diagnosing AKI differed among the included studies, contributing to methodological heterogeneity. Finally, due to insufficient data in the included studies, we were unable to evaluate a potential dose-response relationship between VPT exposure and AKI risk. Future research should investigate whether high-dose vancomycin, particularly when combined with piperacillin/tazobactam, further amplifies the risk of kidney injury.

## Conclusion

This study demonstrates a significantly elevated risk of AKI associated with VPT combination therapy compared to vancomycin combine with cefepime or meropenem, piperacillin/tazo-bactam or vancomycin monotherapy. These findings underscore the critical importance of judicious selection of combination antibiotic regimens and vigilant monitoring of renal function, particularly in critically ill patients who are already at heightened risk for AKI. It is urgently essential to balance the need for effective antimicrobial therapy against the potential for nephrotoxicity. Future high-quality prospective studies are essential to confirm these findings and explore the potential mechanism contributing these associations.

## Data Availability

The original contributions presented in the study are included in the article/**Supplementary Material**. Further inquiries can be directed to the corresponding author.
