# Supplementary material for: Association between concomitant use of vancomycin-piperacillin/tazobactam and acute kidney injury in real-world setting
Source: Front Physiol. 2026 May 21;17:1793497. doi: 10.3389/fphys.2026.1793497 (PMC13233363; doi:10.3389/fphys.2026.1793497)
Supplement: Supplementary file 1 [file DataSheet1.docx]

SUPPLEMENTAL FILE

Title: Association between Concomitant use of Vancomycin-Piperacillin/Tazobactam and Acute Kidney Injury in Real-World Setting

Contents

**Tables**

[eTable 1. Search strategy to identify studies reporting the incidence of acute kidney injury in patients with vancomycin plus piperacillin-tazobactam... 3](#_Toc42611083)

[eTable 2. Quality assessment scale 4](#_Toc42611084)

[eTable 3. Excluded studies with reasons 6](#_Toc42611084)

[eTable 4. Detailed demographics and clinical characteristics of the included studies 7](#_Toc42611086)

[eTable 5. Quality scores of the included studies 9](#_Toc42611087)

[eTable 6. Sensitivity analysis of AKI rate in patients with antimicrobial therapy 1](#_Toc42611088)1

[eTable 7. Meta-regression of the risk of AKI events in patients with antimicrobial therapy 1](#_Toc42611091)3

**Figures**

[eFigure 1. Incidence of AKI in patients with vancomycin+ piperacillin-tazobactam therapy 1](#_Toc42611099)4

[eFigure 2. Incidence of AKI in patients by clinical setting with vancomycin + piperacillin-tazobactam therapy 1](#_Toc42611099)5

[eFigure 3. Incidence of AKI in patients by region with vancomycin + piperacillin-tazobactam therapy 1](#_Toc42611097)6

[eFigure 4. Incidence of AKI in patients with vancomycin monotherapy 1](#_Toc42611099)7

[eFigure 5. Incidence of AKI by region in patients with vancomycin monotherapy 1](#_Toc42611099)8

[eFigure 6. Incidence of AKI by clinical setting in patients with vancomycin monotherapy 1](#_Toc42611099)9

[eFigure 7. Incidence of AKI in patients with piperacillin-tazobactam monotherapy 2](#_Toc42611099)0

[eFigure 8. Incidence of AKI in patients by region with piperacillin-tazobactam monotherapy 2](#_Toc42611098)0

[eFigure 9. Incidence of AKI in patients by clinical setting with piperacillin-tazobactam monotherapy 2](#_Toc42611099)1

[eFigure 10. Incidence of AKI in patients with vancomycin+cefepime 2](#_Toc42611099)2

[eFigure 11. Incidence of AKI by clinical setting in patients with vancomycin+cefepime 2](#_Toc42611099)3

[eFigure 12. Incidence of AKI in patients with vancomycin+meropenem 2](#_Toc42611099)4

[eFigure 13. Incidence of AKI by region in patients with vancomycin+meropenem 2](#_Toc42611099)5

[eFigure 14. Incidence of AKI by clinical setting in patients with vancomycin+meropenem 2](#_Toc42611099)6

[eFigure 15. Funnel plot of incidence of AKI in patients with vancomycin+piperacillin-tazobactam 2](#_Toc42611099)7

[eFigure 16. Funnel plot of incidence of AKI in patients with vancomycin+cefepime 2](#_Toc42611099)7

[eFigure 17. Funnel plot of incidence of AKI in patients with vancomycin+meropenem 2](#_Toc42611099)8

[References 2](#_Toc42611100)9

# eTable 1. Search strategy to identify studies reporting the incidence of acute kidney injury in patients with vancomycin plus piperacillin-tazobactam

| **Literature databases** | **Search items** | **Items found** |
| --- | --- | --- |
| **MEDLINE via Pubmed** | #1: “Vancomycin”[MeSH Terms] OR “Vancomycin”[Title/Abstract] OR “Piperacillin-Tazobactam” [Title/Abstract] OR “ Cefepime ”[Title/Abstract] OR “Meropenem”[Title/Abstract]  #2 : “AKI”[MeSH Terms] OR “AKI”[Title/Abstract] OR “acute kidney injury” [MeSH Terms] OR “acute kidney injury”[Title/Abstract] OR “nephrotoxicity”[MeSH Terms] OR “nephrotoxicity”  #1 AND #2 | 291 |
| **EMBASE** | #1: ‘Vancomycin’/exp OR ‘Vancomycin’:ti,ab,kw OR ‘Piperacillin-Tazobactam’: ti,ab,kw OR ‘Piperacillin-Tazobactam’ /exp OR ‘Cefepime’: ti,ab,kw OR ‘Meropenem’: ti,ab,kw  #2: ‘AKI’/exp OR ‘AKI’:ti,ab,kw OR ‘acute kidney injury’/exp OR ‘acute kidney injury’: ti,ab,kw OR ‘nephrotoxicity’/exp OR nephrotoxicity’: ti,ab,kw  #1 AND #2 | 55 |
| **COCHRANE** | #1: MeSH descriptor: [Vancomycin] OR Vancomycin: ti,ab,kw OR Piperacillin-Tazobactam: ti,ab,kw OR Meropenem: ti,ab,kw OR Cefepime: ti,ab,kw  #2: MeSH descriptor: [AKI] OR AKI: ti,ab,kw OR MeSH descriptor: [acute kidney injury] OR acute kidney injury: ti,ab,kw OR nephrotoxicity: ti,ab,kw  #1 AND #2 | 28 |
| **Overall** |  | 374 |
| **Duplication** |  | 25 |

**eTable 2. Quality assessment scale**

| **Bias type** | **Selection**  **(sample population)** | **Selection**  **(sample size)** | **Selection (participation rate)** | **Performance bias (outcome assessment)** | **Performance bias (analytical methods to control for bias)** |
| --- | --- | --- | --- | --- | --- |
| **Low risk**  **(score=2)** | 1) Sample from the general population, not a select group;  2) Consecutive unselected population;  3) Rationale for case and control selection explained. | 1) Sample size calculation performed and adequate. | 1) High response rate (>85%). | 1) Diagnosis using consistent criteria and direct examination. | 1) Analysis appropriate for the type of sample (subgroup analysis/regression etc.) |
| **Moderate risk (score=1)** | 1) Sample selected from large population but selection criteria not defined;  2) Sample selection ambiguous but may be representative;  3) Rationale for cases and controls not explained;  4) Eligibility criteria not explained;  5) Analysis to adjust for sampling strategy bias. | 1) Sample size calculation performed and reasons for not meeting sample size given;  2) Sample size calculation not performed but all eligible persons studied. | 1) Moderate response rate (70-85%). | 1) Assessment from administrative database or register;  2) Assessment from hospital record or interviewer. | 1) Analysis does not account for common adjustment. |
| **High risk (score=0)** | 1) Highly select population making it difficult to generalise finding;  2) Sample selection ambiguous and sample unlikely to be representative. | 1) Sample size estimation unclear or only sub-sample studied. | 1) Low response rate (<70%);  2) Response rate not reported. | 1) Assessment from non-validated data or generic estimate from the overall population. | 1) Data confusing. |

#

# eTable 3. Excluded studies with reasons

| **Excluded Studies** | **Reason for exclusion** |
| --- | --- |
| Zhang, 2024^[1](#_ENREF_1)^ | Children data |
| Wallis,2018^[2](#_ENREF_2)^ | Review |
| Pan, 2025^[3](#_ENREF_3)^ | Review |
| Kalligeros,2019^[4](#_ENREF_4)^ | Children data |
| Hammond, 2017^[5](#_ENREF_5)^ | Review |
| Giuliano, 2016^[6](#_ENREF_6)^ | Review |
| Covert, 2020^[7](#_ENREF_7)^ | No outcome data |
| Ciarambino, 2020^[8](#_ENREF_8)^ | No outcome data |
| Chen,2018^[9](#_ENREF_9)^ | No specific antibacterial drugs data |
| Blair, 2021^[10](#_ENREF_10)^ | No specific antibacterial drugs data |
| Bellos,2020^[11](#_ENREF_11)^ | No outcome data |
| Alshehri, 2025^[12](#_ENREF_12)^ | No specific antibacterial drugs data |
| Alaradi, 2025^[13](#_ENREF_13)^ | No outcome data |
| Yamashita, 2021^[14](#_ENREF_14)^ | No outcome data |
| Wuerger, 2023^[15](#_ENREF_15)^ | No specific antibacterial drugs data |
| Venugopalan, 2024^[16](#_ENREF_16)^ | No outcome data |
| Tomazini, 2024^[17](#_ENREF_17)^ | No outcome data |
| Su, 2023^[18](#_ENREF_18)^ | No specific antibacterial drugs data |

# eTable 4. Detailed demographics and clinical characteristics of the included studies

| **Study name** | **Total number** | **Age** | **Female** | **BMI** | **Cancer** | **Hypertension** | **DM** | **CVD** | **CrCl** | **Renal toxic medications** | **Vancomycin concentration** | **Impact**  **on AKI** | **Time to AKI, days** |
| --- | --- | --- | --- | --- | --- | --- | --- | --- | --- | --- | --- | --- | --- |
| Burgess, 2014 | 191 | 58.30 | 51.20 | NR | NR | NR | NR | NR | NR | None | NR | NR | NR |
| Gomes, 2014 | 224 | 52.40 | 41.10 | NR | 17.90 | 58.90 | 38.40 | 30.40 | NR | None | NR | NR | NR |
| Navalkele, 2016 | 558 | 56.50 | 55.00 | 26.00 | 18.00 | 63.00 | NR | 42.00 | NR | None | NR | NR | NR |
| Rutter, 2016 | 4193 | 49.60 | 39.60 | NR | NR | 26.20 | NR | NR | NR | None | NR | NR | NR |
| Kim, 2015 | 228 | 48.70 | 35.10 | 26.80 | 7.00 | 39.90 | 49.00 | NR | 74.30 | None | NR | NR | NR |
| Meaney, 2014 | 125 | 50.90 | 50.40 | 25.90 | NR | 44.80 | 20.80 | NR | 84.60 | None | NR | NR | NR |
| Rutter, 2017 | 11650 | 52.50 | 52.10 | NR | NR | NR | NR | NR | NR | Acyclovir,  ARB | NR | NR | 5.00 |
| Al Yamia, 2017 | 183 | 52.40 | 34.30 | NR | 17.60 | 40.70 | 17.60 | NR | NR | Acyclovir,  ARB | NR | NR | NR |
| Hammond, 2016 | 122 | 55.40 | 49.00 | NR | 4.10 | 38.80 | 22.40 | 14.30 | NR | None | NR | NR | NR |
| Moenster, 2014 | 139 | 62.80 | NR | NR | NR | NR | NR | NR | 72.00 | None | 15.8mg/kg | None | NR |
| Peyko, 2017 | 85 | 74.60 | 51.80 | NR | NR | NR | 37.60 | NR | 51.60 | Yes | NR | NR | NR |
| Petite, 2016 | 417 | 62.00 | 49.10 | NR | NR | NR | NR | NR | NR | None | NR | NR | NR |
| Anderson, 2017 | 455 | 57.50 | NR | 28.90 | NR | NR | NR | NR | 110.00 | NR | NR | NR | NR |
| Balci, 2018 | 402 | 52.40 | 48.60 | NR | 18.60 | 44.30 | 21.40 | 21.40 | NR | Yes | NR | NR | NR |
| Carreno, 2018 | 142 | 56.00 | 52.10 | NR | NR | NR | NR | NR | 76.00 | None | NR | NR | NR |
| Jeon, 2017 | 5335 | 65.10 | 41.87 | NR | NR | 25.30 | 16.57 | NR | NR | None | NR | NR | NR |
| Cannon, 2017 | 266 | 65.30 | 51.20 | NR | NR | NR | 42.10 | NR | NR | None | NR | NR | NR |
| Robertson, 2018 | 169 | 55.70 | 42.40 | 29.50 | NR | 45.90 | 36.50 | 15.30 | 77.30 | Amphotericin | 25.4mg/kg | NR | NR |
| Mullins, 2018 | 141 | 67.10 | 60.60 | NR | NR | NR | 37.20 | 35.10 | NR | None | 14.9mg/kg | NR | NR |
| Blevins, 2019 | 758 | 56.70 | 51.60 | NR | NR | NR | 23.50 | NR | NR | Acyclovir  ACE inhibitor | NR | NR | 3.2 |
| Ide,2019 | 141 | 70.70 | 37.50 | NR | 43.60 | NR | 23.60 | NR | 62.40 | ACE inhibitors  Amphotericin | NR | NR | NR |
| Kang, 2019 | 157 | 59.10 | 33.90 | 23.00 | NR | 85.00 | 24.60 | NR | 97.80 | None | NR | NR | NR |
| Schreier, 2019 | 1926 | 55.70 | 42.40 | 29.50 | NR | 45.90 | 36.50 | 22.40 | NR | Amphotericin  Aminoglycoside | 25.4mg/kg | NR | NR |
| Liu, 2021 | 526 | 56.88 | 35.10 | NR | 37.60 | 28.80 | 10.60 | 12.40 | 62.10 | Diuretic  NSAID | 10.7mg/kg | NR | NR |
| Rungkitwattanakul, 2022 | 207 | 71.50 | 54.10 | NR | NR | NR | NR | NR | NR | None | NR | NR | 4.00 |
| Tookhi, 2021 | 158 | 56.00 | 55.80 | NR | NR | NR | NR | NR | NR | Foscarnet  Loop diuretic | NR | NR | NR |
| Wu, 2024 | 349 | 61.80 | 38.30 | NR | 18.18 | 36.36 | 29.22 | 22.08 | NR | ACEI/ARB  Vasopressors | NR | NR | 3.00 |
| Molina, 2020 | 394 | 55.00 | 32.60 | 10.50 | 8.10 | 25.60 | 19.30 | NR | 117.00 | ACE inhibitor  Acyclovir | NR | NR | NR |
| Whitenack, 2022 | 480 | 57.00 | 45.10 | NR | NR | 61.10 | 34.70 | 34.00 | NR | None | NR | NR | NR |
| Miano, 2022 | 739 | 60.60 | 45.70 | NR | NR | 52.30 | 26.00 | NR | NR | ACE inhibitor  Acyclovir | NR | NR | NR |
| Chen, 2023 | 3648 | 66.20 | 48.10 | NR | NR | NR | NR | NR | NR | NSAIDS | NR | NR | NR |
| Piccuirro, 2021 | 210 | 54.90 | 41.40 | NR | NR | 11.40 | NR | NR | NR | None | NR | NR | NR |
| Komerdelj, 2022 | 3199 | 56.00 | 34.00 | 27.50 | 13.40 | 49.00 | 49.50 | NR | NR | None | NR | NR | 4.2 |
| Inage, 2020 | 593 | 64.80 | 33.20 | NR | NR | 30.20 | NR | NR | NR | None | NR | NR | NR |
| Buckley, 2022 | 1044 | 62.00 | 39.50 | 27.50 | 22.60 | 48.40 | 38.90 | NR | NR | None | NR | NR | 3.5 |

BMI: body mass index; DM: diabetes; CVD: cardiovascular disease; NR: not reported.

#

# eTable 5. Quality scores of the included studies

| **Study** | **Sample population** | **Sample size** | **Participation rate** | **Outcome assessment** | **Analytical methods to control for bias** | **Total score** |
| --- | --- | --- | --- | --- | --- | --- |
| Burgess, 2014 | 2 | 2 | 2 | 1 | 2 | 9 |
| Gomes, 2014 | 2 | 2 | 2 | 1 | 1 | 8 |
| Navalkele, 2016 | 2 | 2 | 1 | 1 | 1 | 7 |
| Rutter, 2016 | 2 | 1 | 1 | 1 | 1 | 6 |
| Kim, 2015 | 2 | 2 | 2 | 1 | 2 | 9 |
| Meaney, 2014 | 2 | 2 | 2 | 1 | 1 | 8 |
| Rutter, 2017 | 2 | 2 | 2 | 1 | 2 | 9 |
| Al Yamia, 2017 | 2 | 2 | 2 | 1 | 2 | 9 |
| Hammond, 2016 | 2 | 2 | 2 | 1 | 1 | 8 |
| Moenster, 2014 | 2 | 2 | 2 | 1 | 1 | 8 |
| Peyko, 2017 | 2 | 2 | 2 | 1 | 2 | 9 |
| Petite, 2016 | 2 | 2 | 2 | 1 | 1 | 8 |
| Anderson, 2017 | 2 | 2 | 2 | 1 | 2 | 9 |
| Balci, 2018 | 2 | 2 | 2 | 1 | 1 | 8 |
| Carreno, 2018 | 2 | 2 | 2 | 1 | 2 | 9 |
| Jeon, 2017 | 2 | 2 | 2 | 1 | 2 | 9 |
| Cannon, 2017 | 2 | 2 | 2 | 1 | 1 | 8 |
| Robertson, 2018 | 2 | 2 | 1 | 1 | 1 | 7 |
| Mullins, 2018 | 2 | 1 | 1 | 1 | 1 | 6 |
| Blevins, 2019 | 2 | 2 | 2 | 1 | 2 | 9 |
| Ide,2019 | 2 | 2 | 2 | 1 | 1 | 8 |
| Kang, 2019 | 2 | 2 | 2 | 1 | 2 | 9 |
| Schreier, 2019 | 2 | 2 | 2 | 1 | 2 | 9 |
| Liu, 2021 | 2 | 2 | 2 | 1 | 1 | 8 |
| Rungkitwattanakul, 2022 | 2 | 2 | 2 | 1 | 1 | 8 |
| Tookhi, 2021 | 2 | 2 | 2 | 1 | 2 | 9 |
| Wu, 2024 | 2 | 2 | 2 | 1 | 1 | 8 |
| Molina, 2020 | 2 | 2 | 2 | 1 | 2 | 9 |
| Whitenack, 2022 | 2 | 2 | 2 | 1 | 1 | 8 |
| Miano, 2022 | 2 | 2 | 2 | 1 | 2 | 9 |
| Chen, 2023 | 2 | 2 | 2 | 1 | 2 | 9 |
| Piccuirro, 2021 | 2 | 2 | 2 | 1 | 1 | 8 |
| Komerdelj, 2022 | 2 | 2 | 2 | 1 | 2 | 9 |
| Inage, 2020 | 2 | 2 | 2 | 1 | 1 | 8 |
| Buckley, 2022 | 2 | 2 | 2 | 1 | 2 | 9 |

# eTable 6. Sensitivity analysis of AKI rate in patients with antimicrobial therapy

| Sensitivity analysis of AKI rate in patients with vancomycin monotherapy | | | | | |
| --- | --- | --- | --- | --- | --- |
| **Study omitted** | **Rate (95%CI)** | **Study omitted** | **Rate (95%CI)** | **Study omitted** | **Rate (95%CI)** |
| Burgess, 2014 | 7.47 (4.84-10.10) | Anderson, 2017 | 6.90 (4.32-9.48) | Liu, 2021 | 8.28 (6.06-10.50) |
| Kim, 2015 | 8.11 (5.62-10.60) | Balci, 2018 | 6.70 (4.71-9.29) | Carreno, 2018 | 7.60 (5.01-10.19) |
| Rutter, 2017 | 7.48 (4.27-10.68) |  |  |  |  |
| **Sensitivity analysis of AKI rate in patients with vancomycin+piperacillin-tazobactam therapy** | | | | | |
| **Study omitted** | **Rate (95%CI)** | **Study omitted** | **Rate (95%CI)** | **Study omitted** | **Rate (95%CI)** |
| Burgess, 2014 | 25.75 (23.22-28.29) | Petite, 2016 | 25.54 (23.00-28.09) | Kang, 2019 | 24.87 (22.40-27.33) |
| Gomes, 2014 | 25.23 (22.71-27.74) | Anderson, 2017 | 25.54 (22.99-28.08) | Schreier, 2019) | 24.64 (22.53-26.74) |
| Navalkele, 2016 | 25.36 (22.83-27.90) | Balci, 2018 | 25.15 (22.65-27.65) | Liu, 2021 | 25.77 (23.24-28.31) |
| Rutter, 2016 | 25.74 (22.98-28.51) | Carreno, 2018 | 25.45 (22.92-27.99) | Rungkitwattanakul, 2022 | 25.58 (23.05-28.11) |
| Kim, 2015 | 25.68 (23.14-28.22) | Jeon, 2017 | 25.76 (23.10-28.42) | Tookhi, 2021 | 25.94 (23.42-28.46) |
| Rutter, 2017 | 25.79 (22.94-28.64) | Cannon, 2017 | 25.50 (22.95-28.06) | Wu, 2024 | 25.55 (23.01-28.09) |
| Al Yamia, 2017 | 26.07 (23.59-28.56) | Robertson, 2018 | 25.74 (23.20-28.27) | Molina, 2020 | 25.37 (22.84-27.91) |
| Hammond, 2016 | 25.34 (22.83-27.86) | Mullins, 2018 | 25.37 (22.84-27.90) | Whitenack, 2022 | 25.54 (22.99-28.09) |
| Moenster, 2014 | 25.38 (22.85-27.91) | Blevins, 2019 | 24.96 (22.51-27.41) | Miano, 2022 | 24.88 (22.44-27.31) |
| Peyko, 2017 | 25.24 (22.73-27.75) | Ide, 2019 | 25.38 (22.87-27.89) | Chen, 2023 | 25.80 (23.17-28.44) |
| Piccuirro, 2021 | 25.20 (22.69-27.72) | Komerdelj, 2022 | 25.85 (23.12-28.20) | Inage, 2020 | 25.66 (23.12-28.20) |
| Buckley, 2022 | 25.64 (23.05-28.23) |  |  |  |  |
| **Sensitivity analysis of AKI rate in patients with piperacillin-tazobactam therapy** | | | | | |
| **Study omitted** | **Rate (95%CI)** | **Study omitted** | **Rate (95%CI)** | **Study omitted** | **Rate (95%CI)** |
| Rutter, 2017 | 14.42 (9.40-19.44) | Balci, 2018 | 11.15 (4.79-17.52) | Carreno, 2018 | 13.48 (6.23-20.73) |
| Petite, 2016 | 10.52 (5.25-15.80) |  |  |  |  |
| **Sensitivity analysis of AKI rate in patients with vancomycin+cefepime therapy** | | | | | |
| **Study omitted** | **Rate (95%CI)** | **Study omitted** | **Rate (95%CI)** | **Study omitted** | **Rate (95%CI)** |
| Gomes, 2014 | 16.88 (12.99-20.76) | Moenster, 2014 | 16.69 (12.90-20.47) | Whitenack, 2022 | 15.79 (12.01-19.57) |
| Navalkele, 2016 | 17.14 (13.19-21.09) | Jeon, 2017 | 16.60 (11.61-21.60) | Miano, 2022 | 14.81 (11.83-17.79) |
| Rutter, 2016 | 17.00 (12.77-21.24) | Rungkitwattanakul, 2022 | 17.16 (13.37-20.95) | Piccuirro, 2021 | 17.41 (13.69-21.12) |
| Hammond, 2016 | 15.69 (12.03-19.35) | Molina, 2020 | 16.04 (12.21-19.87) |  |  |

CI: confidence interval; AKI: acute kidney injury.

.

# eTable 7. Meta-regression of the risk of AKI events in patients with antimicrobial therapy.

| **Variables** | **No. of reported studies** | **β coefficient (95%CI)** | ***P* value** |
| --- | --- | --- | --- |
| Mean age | 35 | 0.026 (-0.071-0.051) | 0.421 |
| Female | 33 | 0.014 (-0.031-0.033) | 0.080 |
| BMI | 10 | 0.061 (-0.777-0.763) | 0.723 |
| Cancer | 12 | 0.010 (-0.051-0.071) | 0.450 |
| Hypertension | 21 | 0.024 (-0.101-0.104) | 0.855 |
| DM | 25 | 0.269 (-3.377-3.44) | 0.741 |
| CVD history | 11 | 1.391 (0.269-19.069) | 0.512 |
| CrCl | 12 | 0.421 (0.157-5.495) | 0.806 |

DM: Diabetes; CVD: cardiovascular disease.


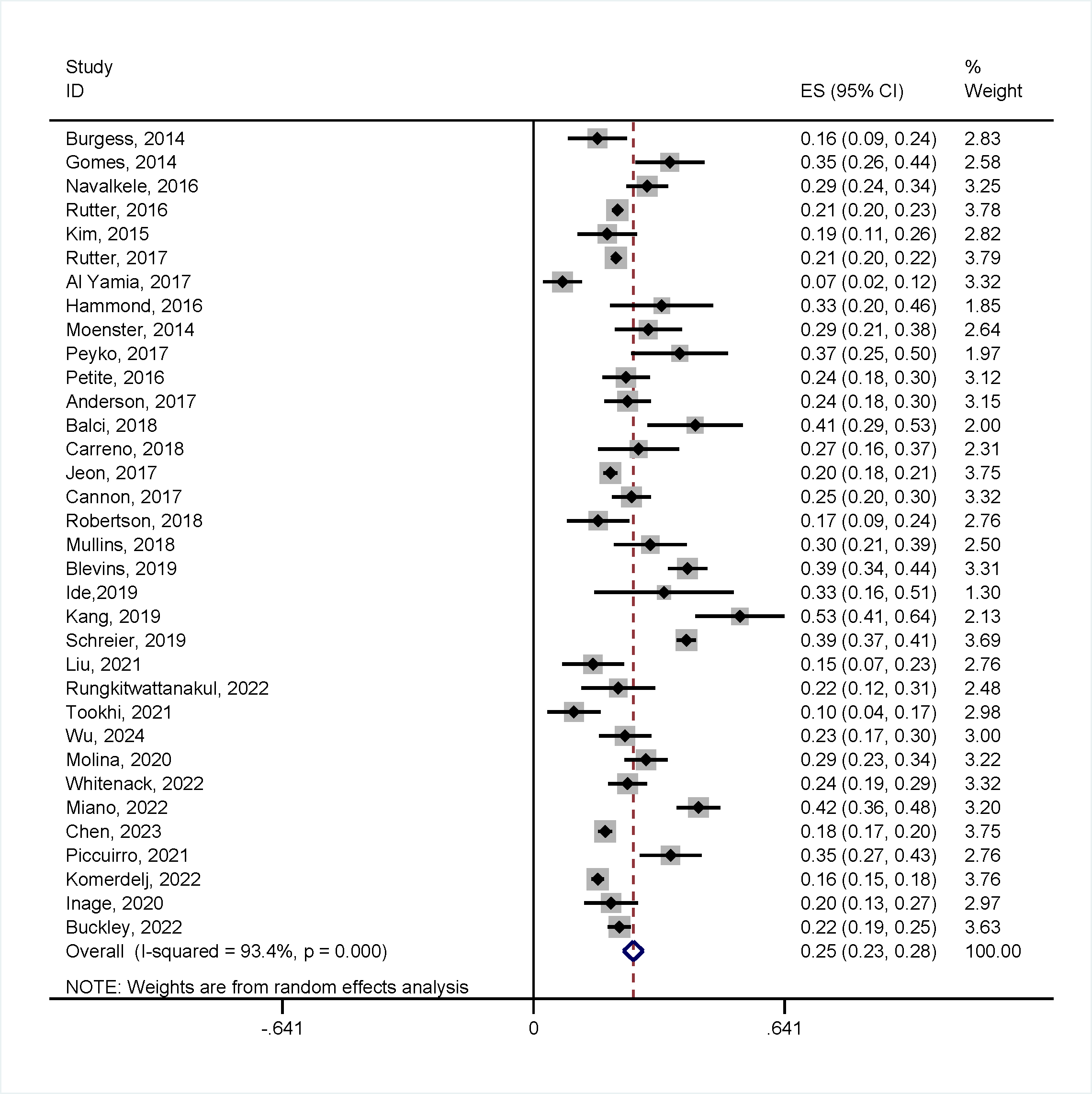


**eFigure 1. Incidence of AKI in patients with vancomycin+ piperacillin-tazobactam therapy**

**
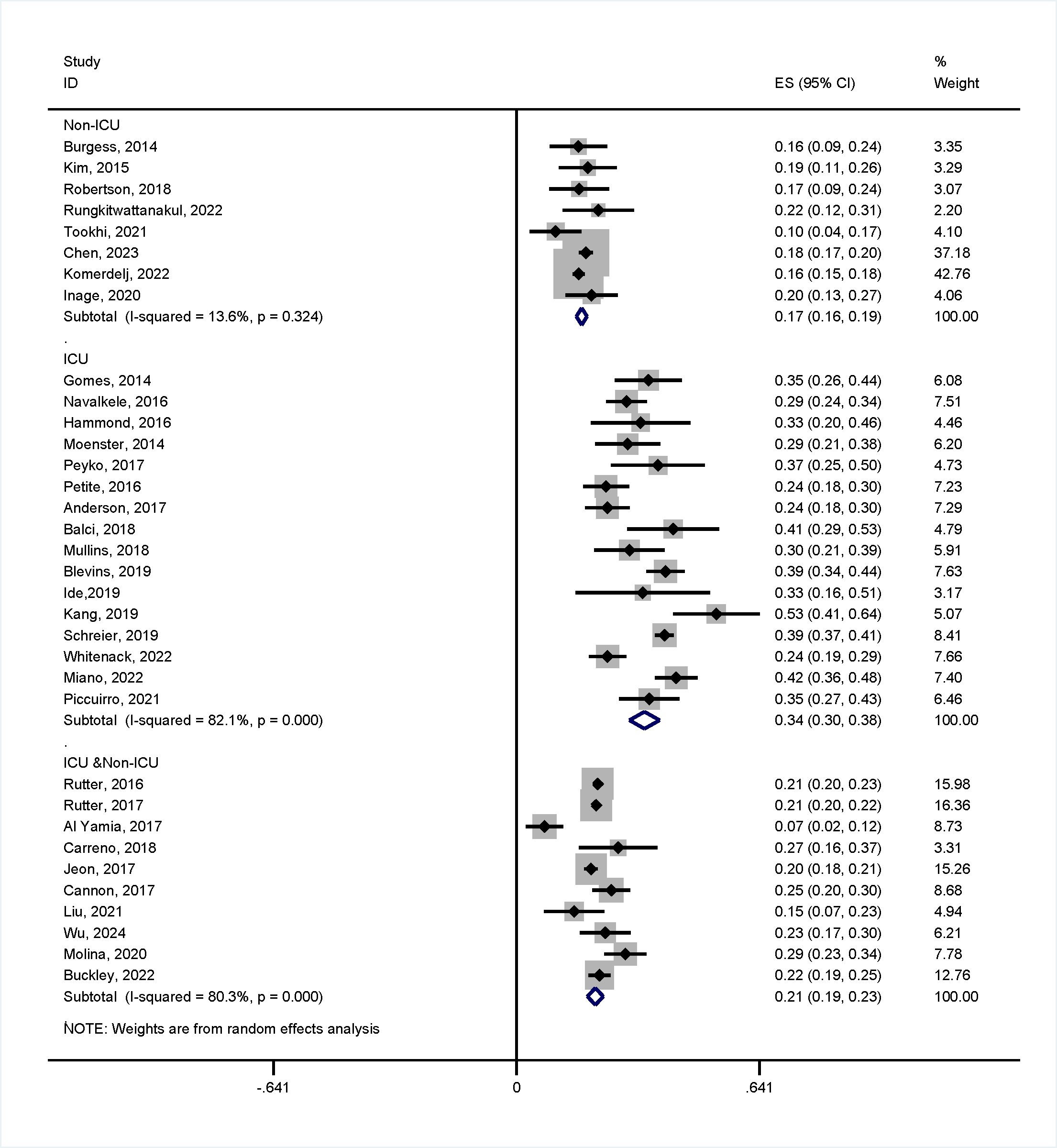
**

**eFigure 2. Incidence of AKI by clinical setting in patients with vancomycin+ piperacillin-tazobactam therapy**

#
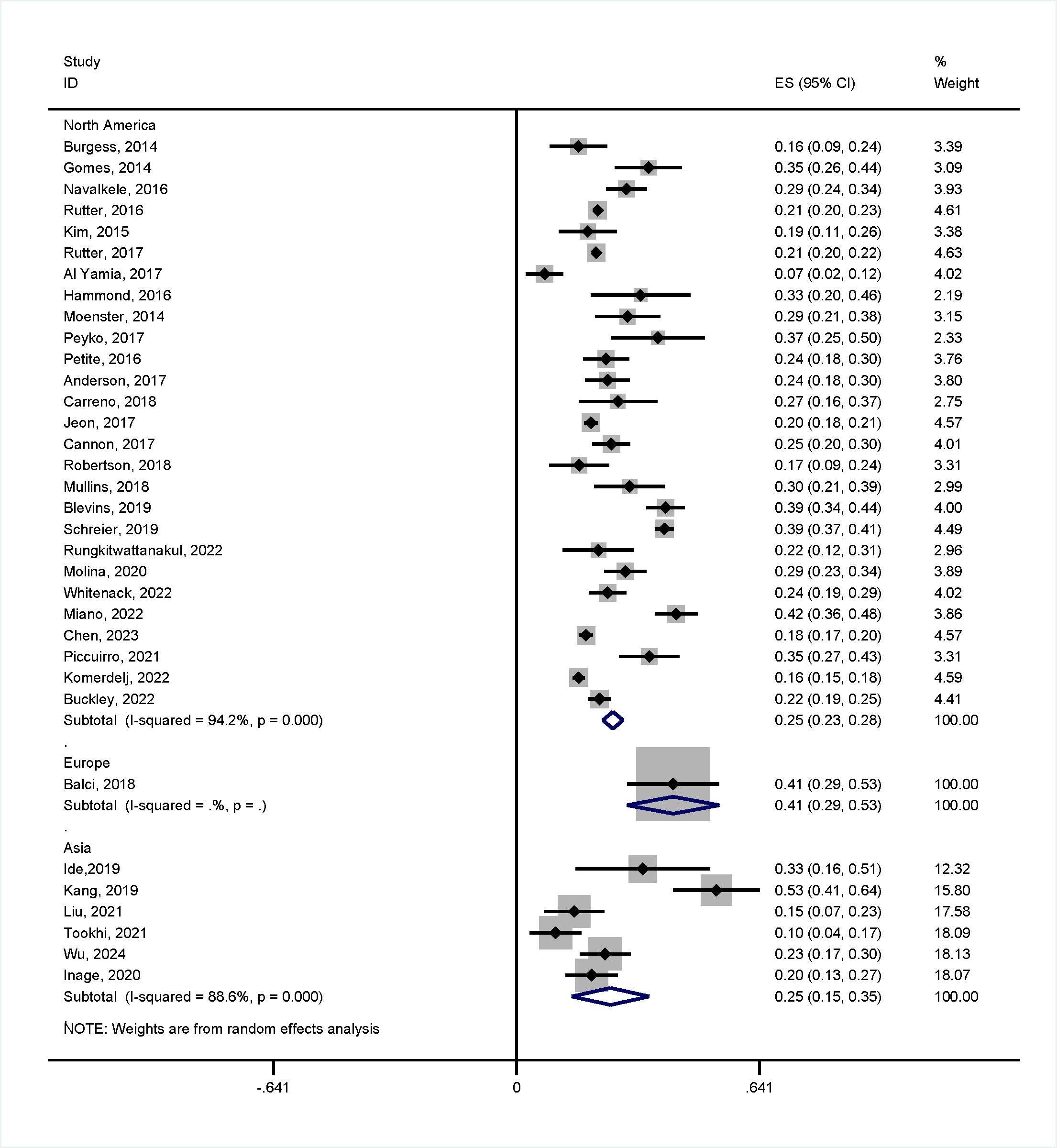


**eFigure 3. Incidence of AKI by region in patients with vancomycin + piperacillin-tazobactam therapy**

**
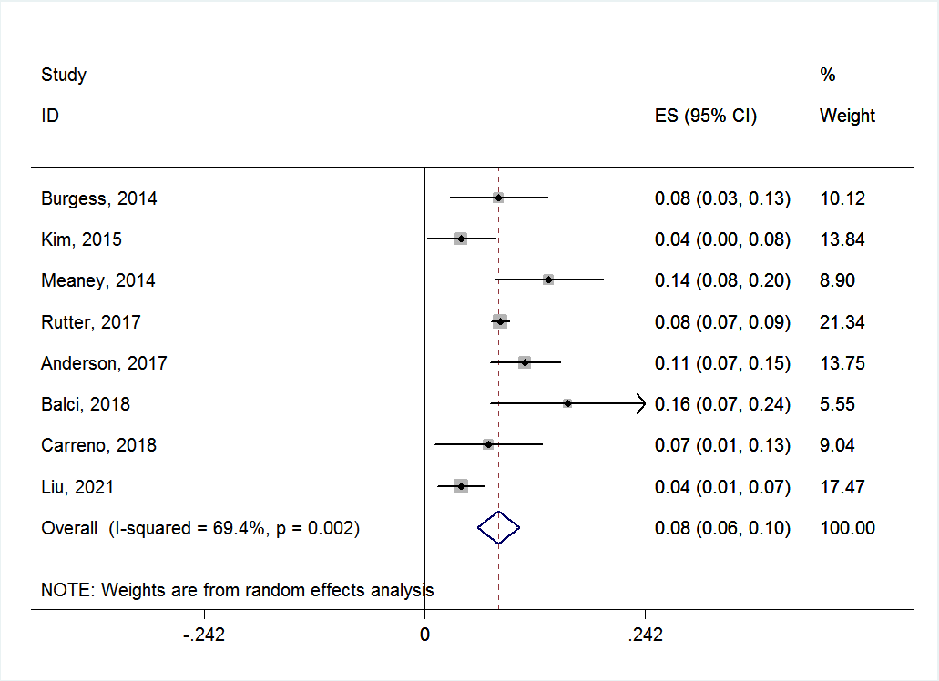
**

**eFigure 4. Incidence of AKI in patients with vancomycin monotherapy**

**
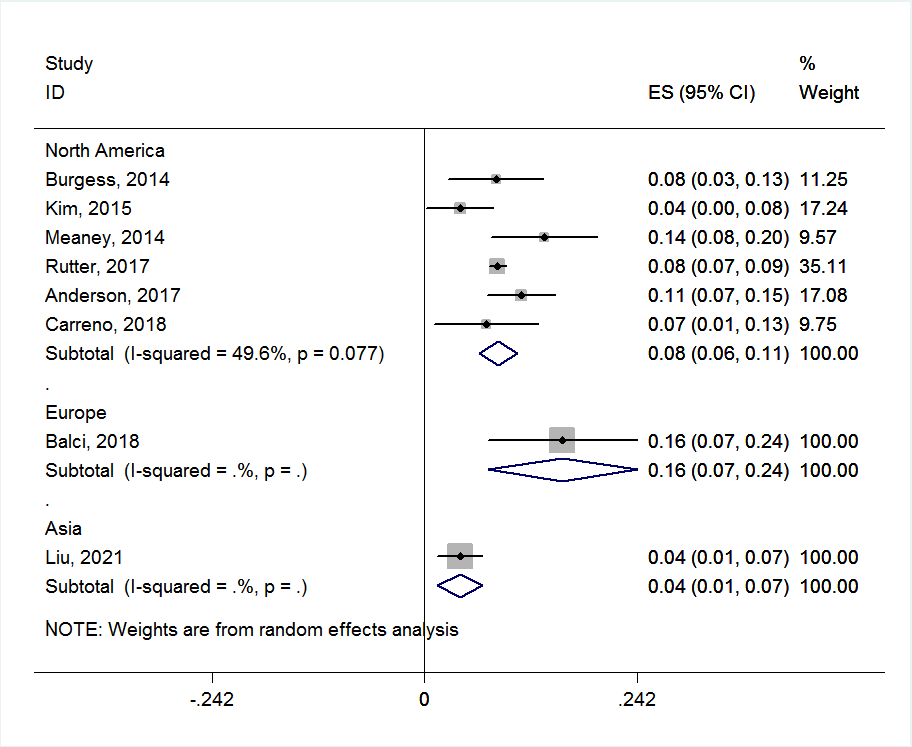
**

**eFigure 5. Incidence of AKI by region in patients with vancomycin monotherapy**


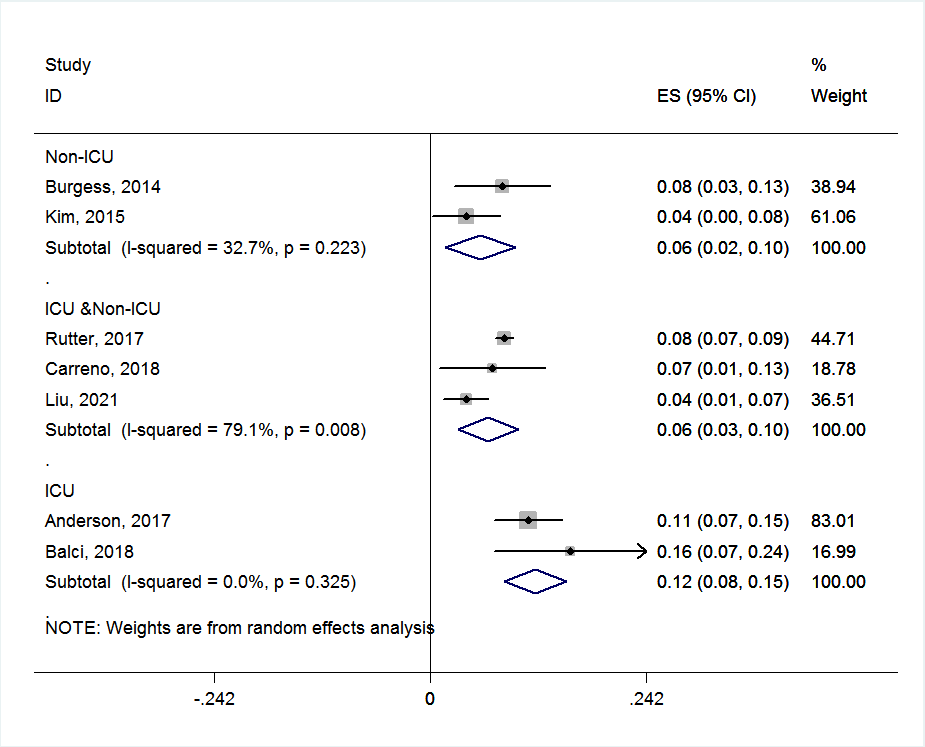


**eFigure 6. Incidence of AKI by clinical setting in patients with vancomycin monotherapy**


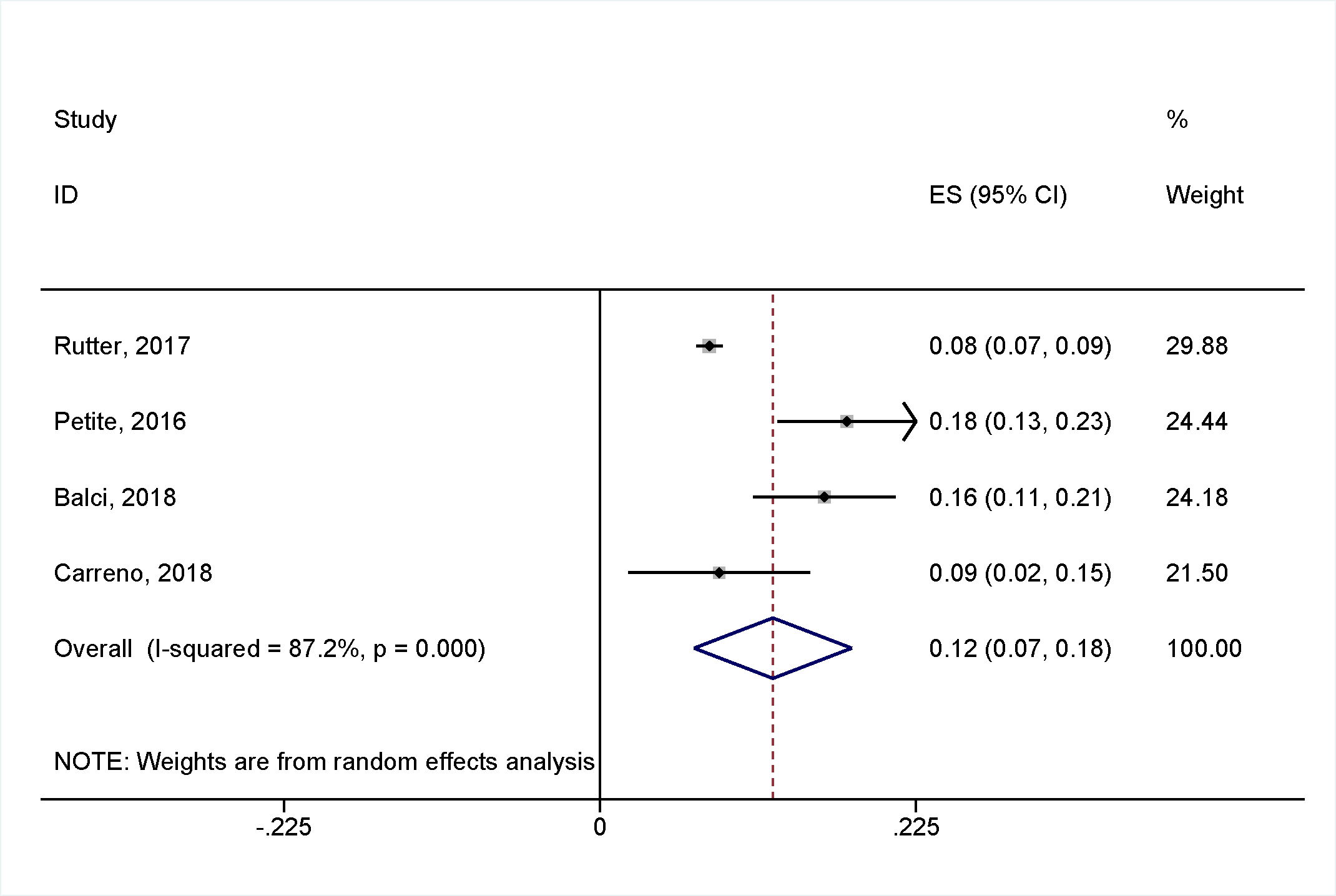


**eFigure 7. Incidence of AKI in patients with piperacillin-tazobactam monotherapy**


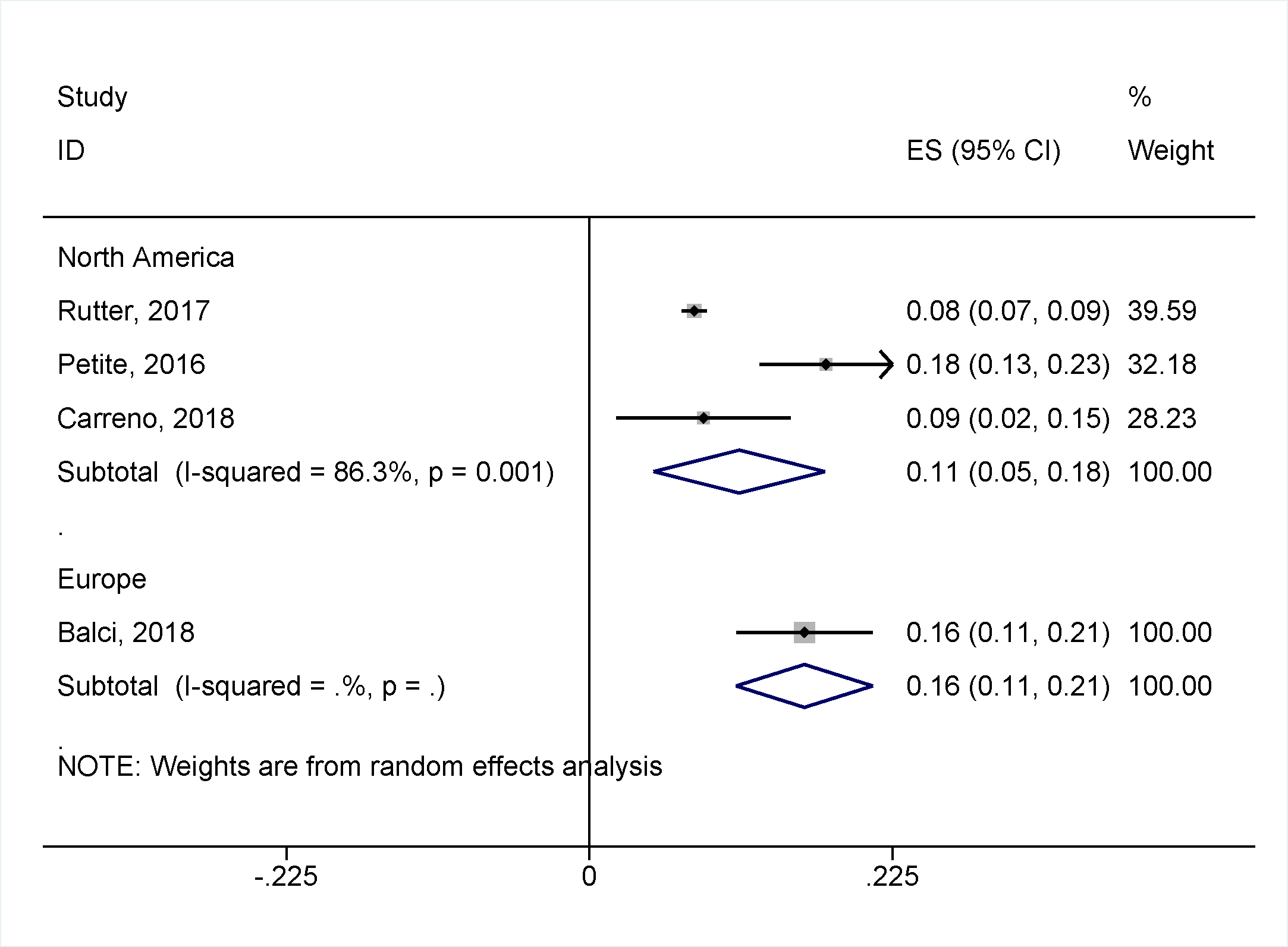


**eFigure 8. Incidence of AKI in patients by region with piperacillin-tazobactam monotherapy**


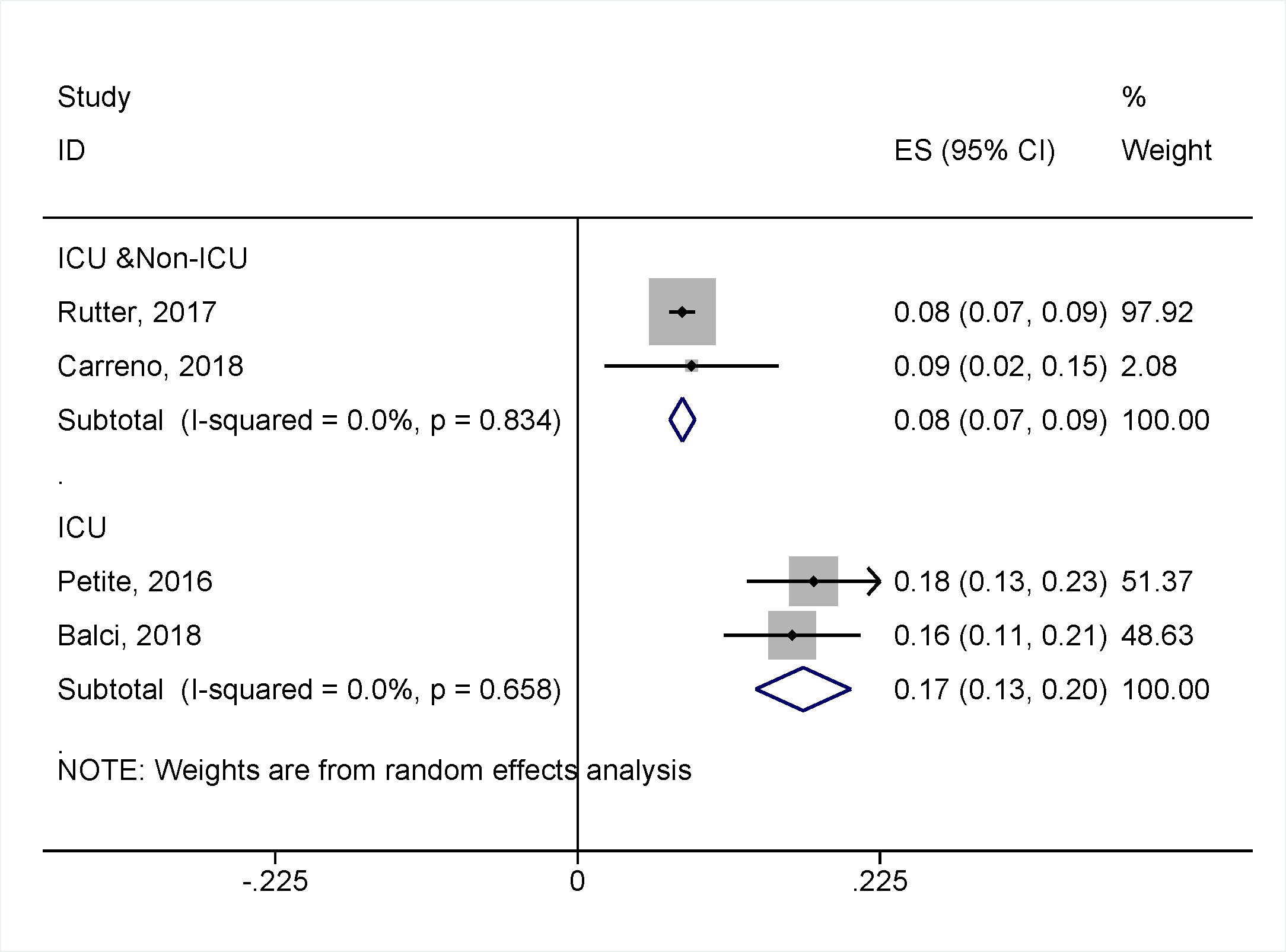


**eFigure 9. Incidence of AKI in patients by clinical setting with piperacillin-tazobactam monotherapy**


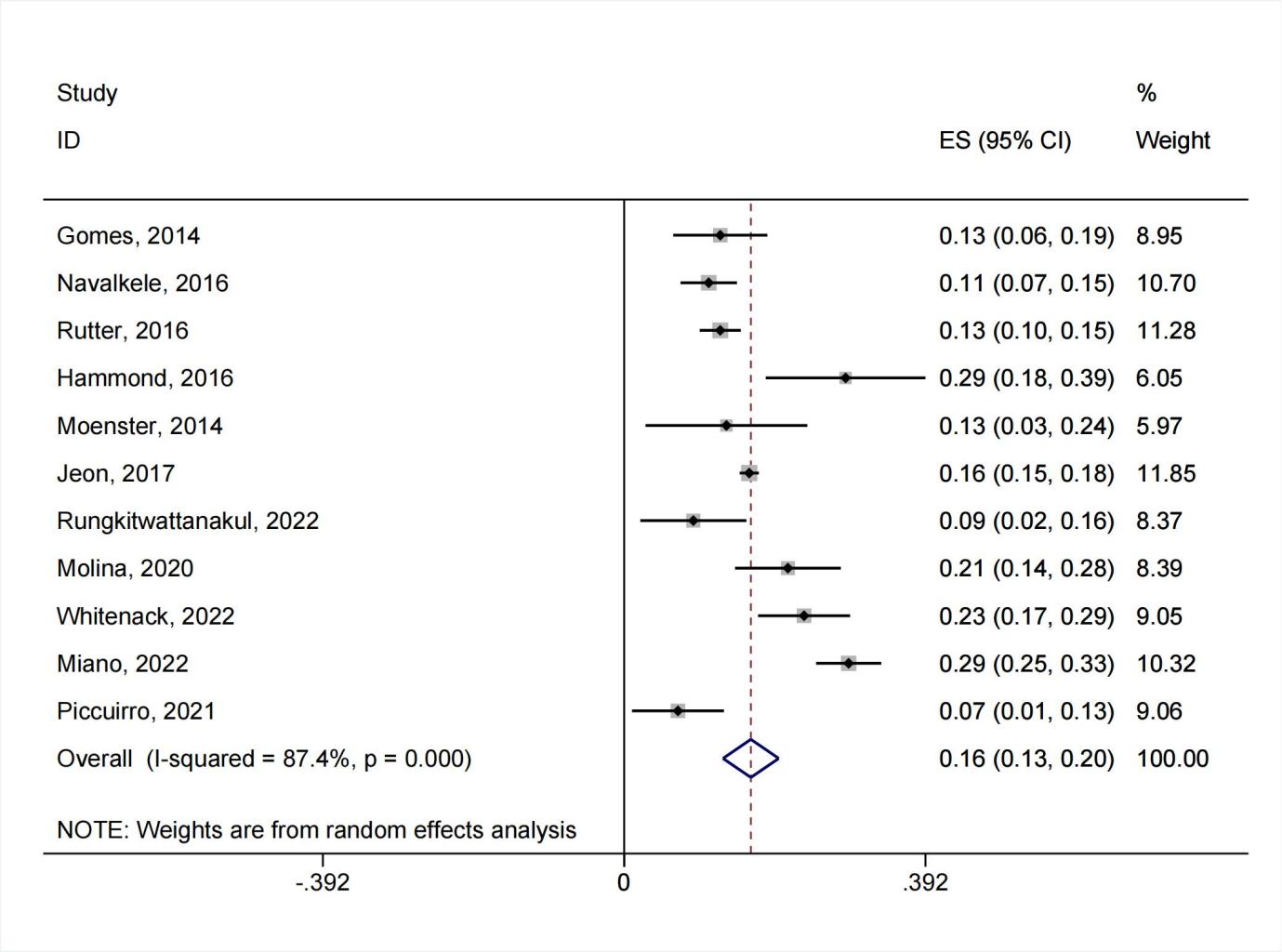


**eFigure 10. Incidence of AKI in patients with vancomycin+cefepime**


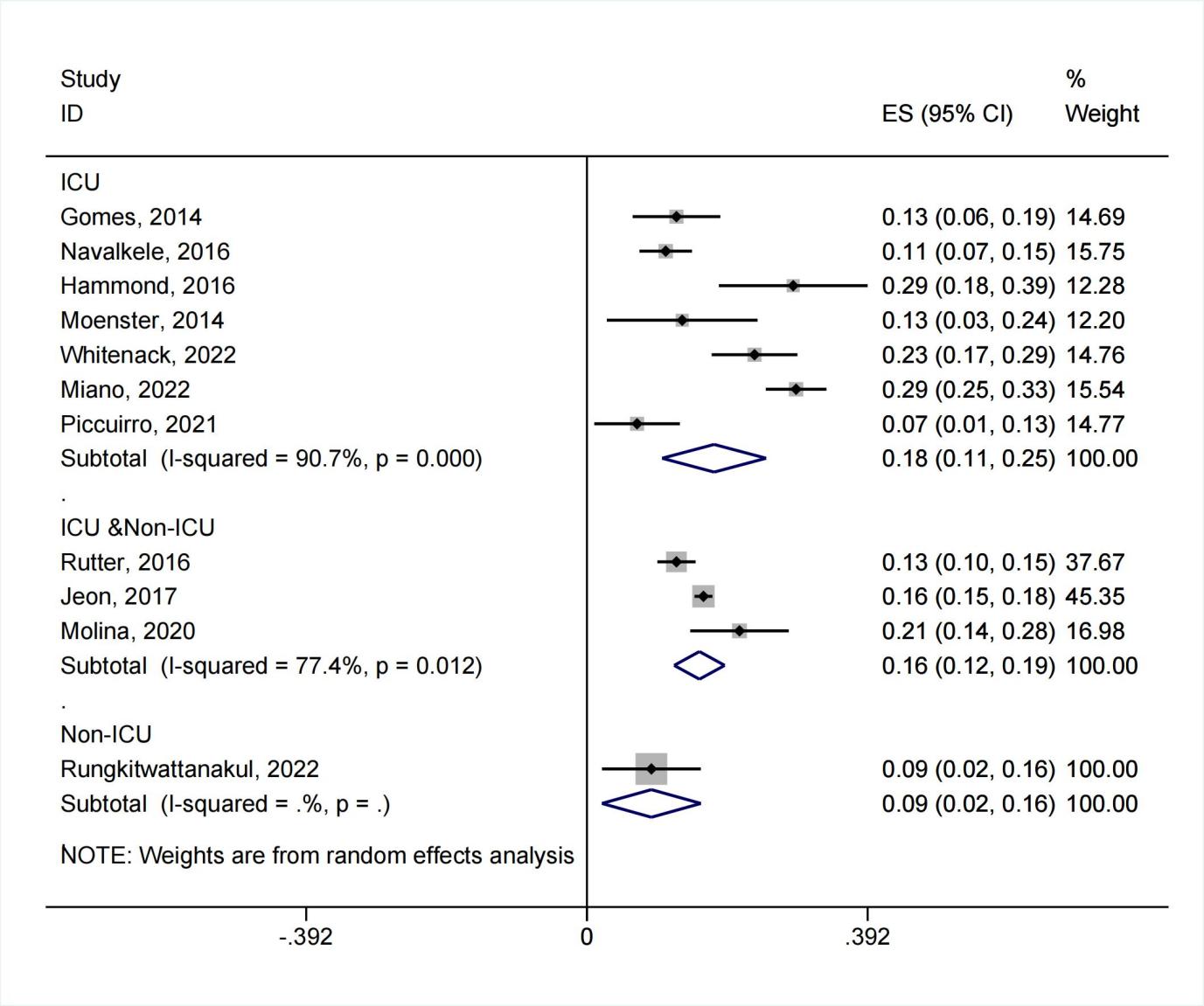


**eFigure 11. Incidence of AKI by clinical setting in patients with vancomycin+cefepime**


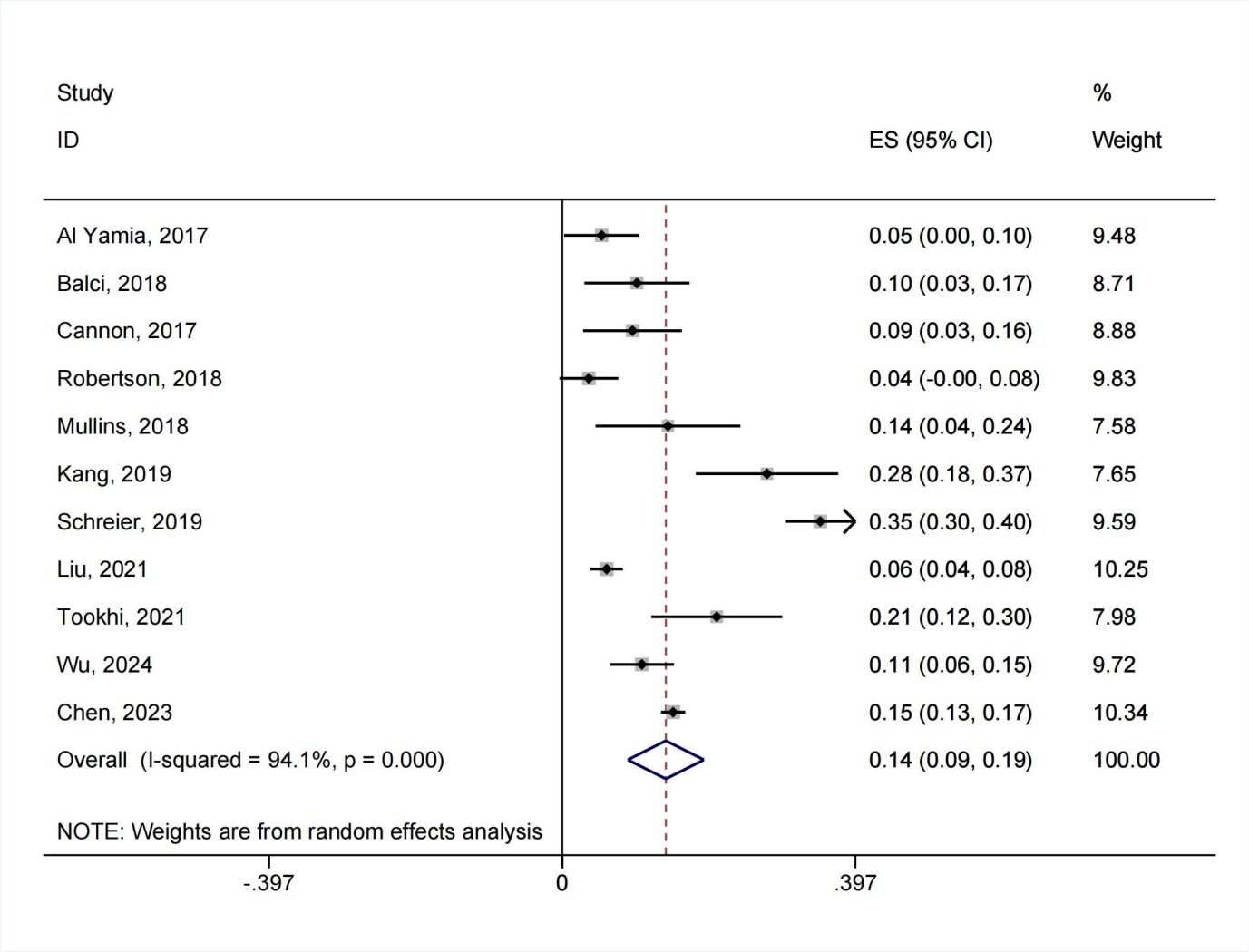


**eFigure 12. Incidence of AKI in patients with vancomycin+meropenem**


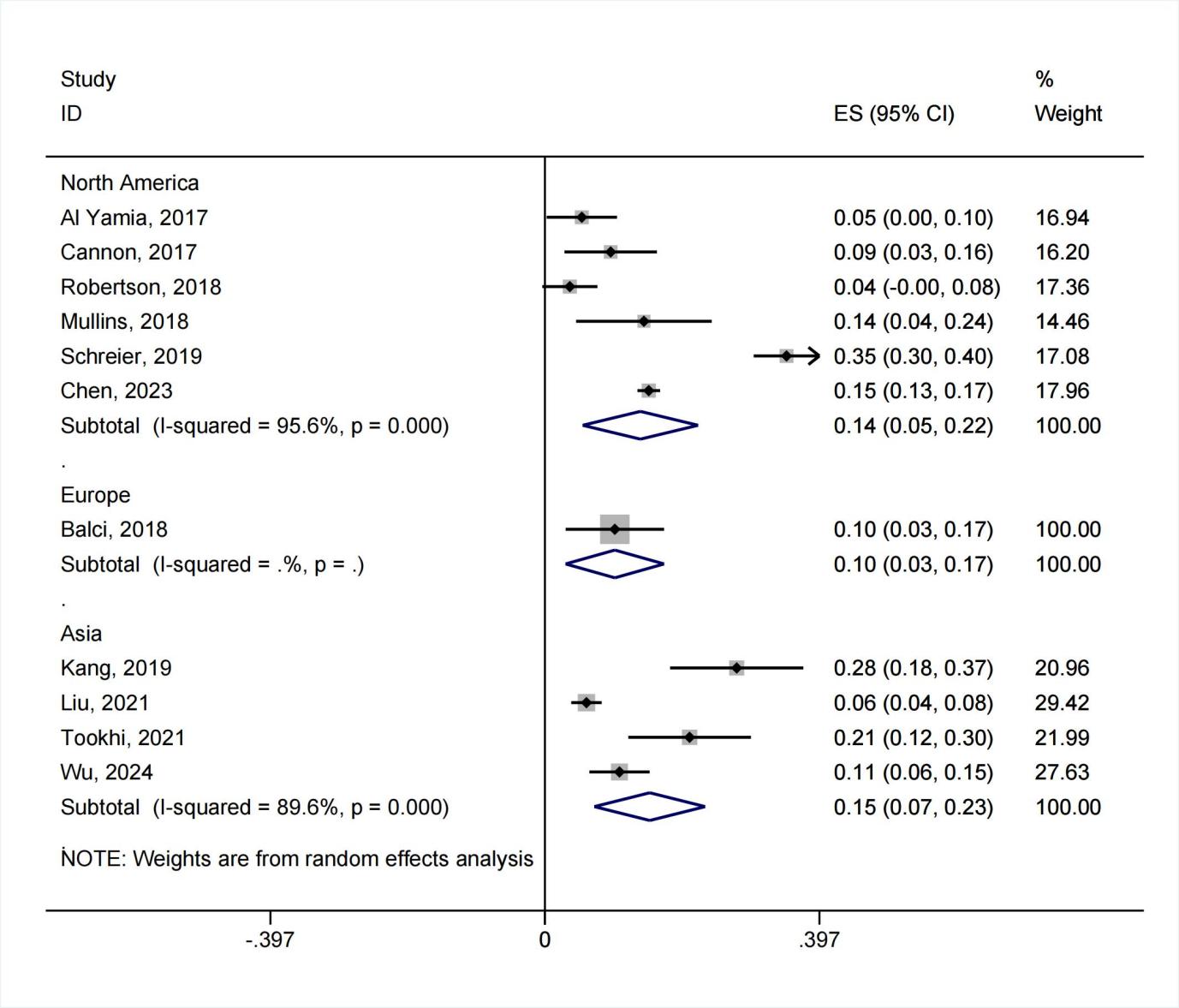


**eFigure 13. Incidence of AKI by region in patients with vancomycin+meropenem**


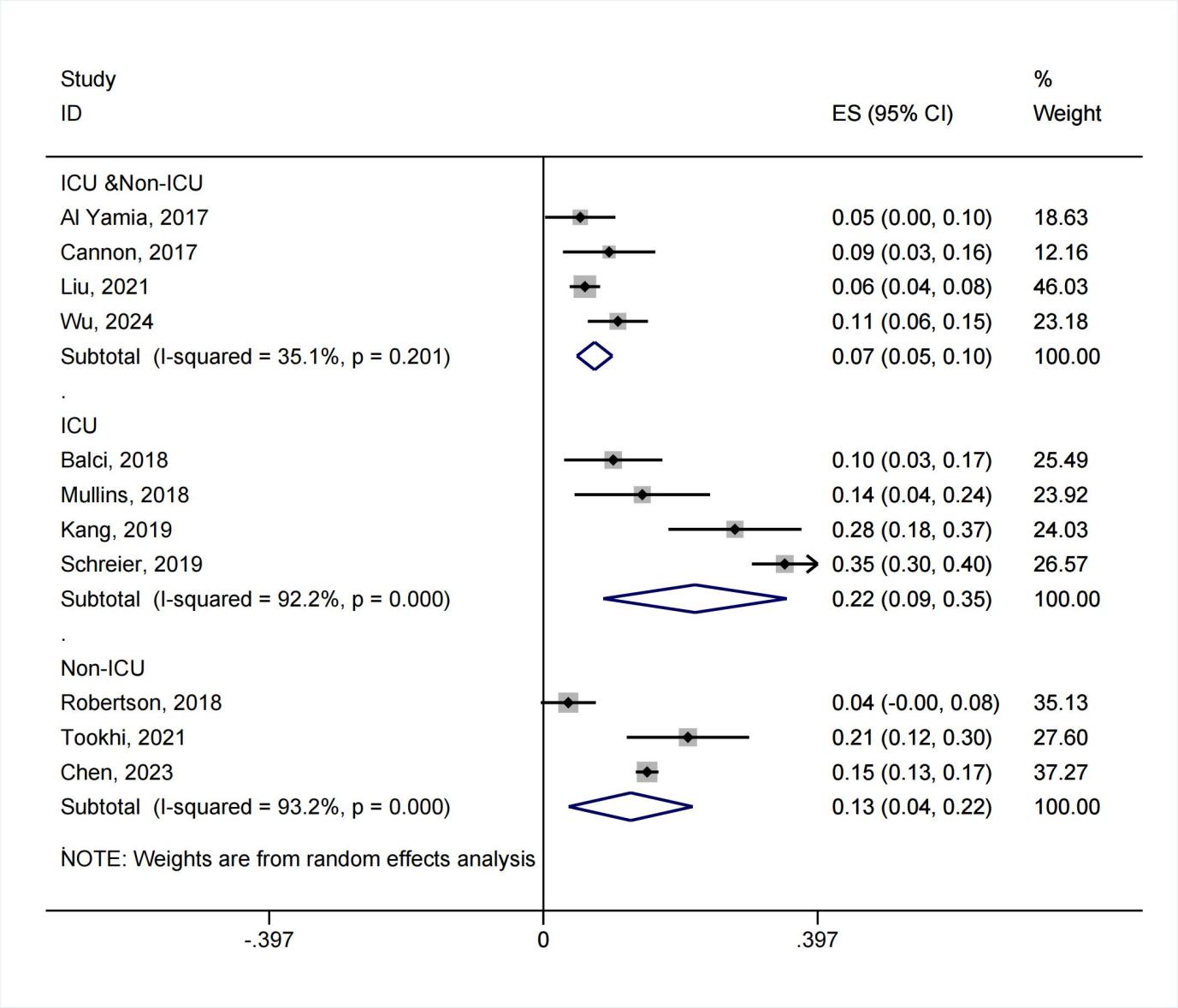


**eFigure 14. Incidence of AKI by clinical setting in patients with vancomycin +meropenem**


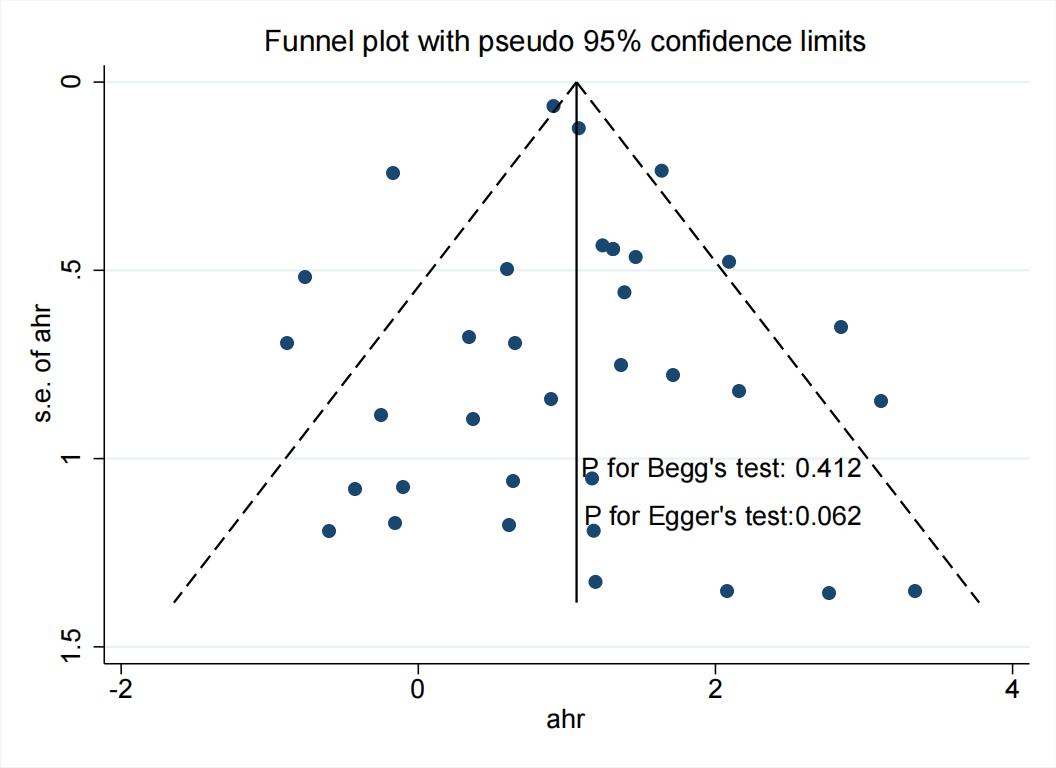


**eFigure 15. Funnel plot of incidence of AKI in patients with vancomycin+ piperacillin-tazobactam**


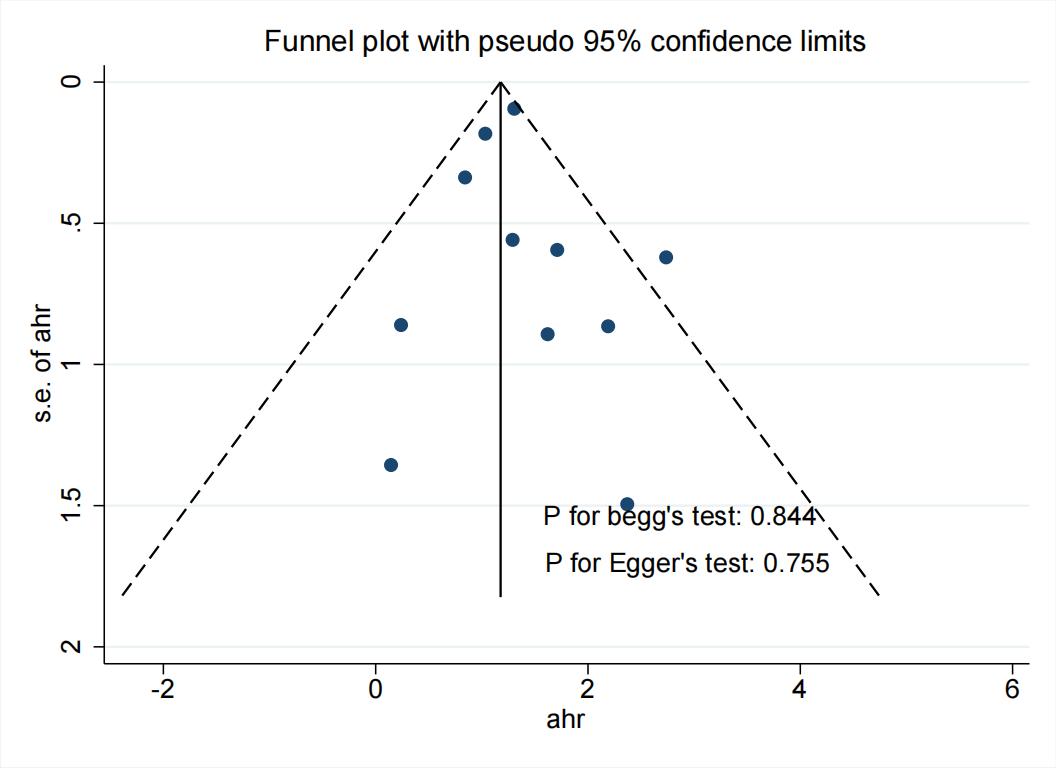


**eFigure 16. Funnel plot of incidence of AKI in patients with vancomycin+cefepime**


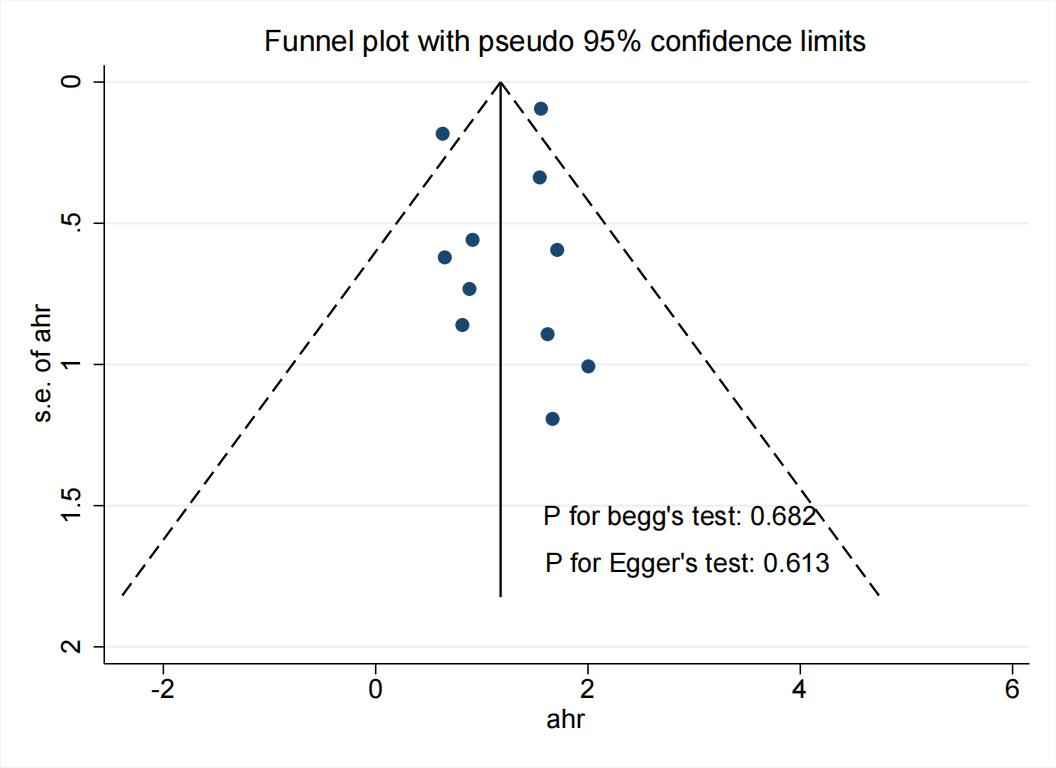


**eFigure 17. Funnel plot of incidence of AKI in patients with vancomycin+meropenem**

# References

1. Zhang M. Epidemiology of vancomycin in combination with piperacillin/tazobactam -associated acute kidney injury in children: A systematic review and meta-analysis. *Antimicrobial agents and chemotherapy*. 2024;58:1034-1044

2. Wallis G, Painter H, Harber M, Collier S, Luther MK, Timbrook TT, Caffrey AR, Dosa D, Lodise TP, LaPlante KL. Vancomycin plus piperacillin-tazobactam and acute kidney injury in adults: A systematic review and meta-analysis. *Transplant infectious disease : an official journal of the Transplantation Society*. 2018;46:12-20

3. Pan K, Li R, Li Y, Ding X, Li X, Lv Q. Vancomycin combined with piperacillin/tazobactam increases the risk of acute kidney injury compared with vancomycin plus other anti-pseudomonal beta-lactams: A systematic review and network meta-analysis. *The Journal of antimicrobial chemotherapy*. 2025;80:47-58

4. Kalligeros M, Karageorgos SA, Shehadeh F, Zacharioudakis IM, Mylonakis E. The association of acute kidney injury with the concomitant use of vancomycin and piperacillin/tazobactam in children: A systematic review and meta-analysis. *Antimicrobial agents and chemotherapy*. 2019;63

5. Hammond DA, Smith MN, Li C, Hayes SM, Lusardi K, Bookstaver PB. Systematic review and meta-analysis of acute kidney injury associated with concomitant vancomycin and piperacillin/tazobactam. *Clinical infectious diseases : an official publication of the Infectious Diseases Society of America*. 2017;64:666-674

6. Giuliano CA, Patel CR, Kale-Pradhan PB. Is the combination of piperacillin-tazobactam and vancomycin associated with development of acute kidney injury? A meta-analysis. *Pharmacotherapy*. 2016;36:1217-1228

7. Covert KL, Knoetze D, Cole M, Lewis P. Vancomycin plus piperacillin/tazobactam and acute kidney injury risk: A review of the literature. *Journal of clinical pharmacy and therapeutics*. 2020;45:1253-1263

8. Ciarambino T, Giannico OV, Campanile A, Tirelli P, Para O, Signoriello G, Giordano M. Acute kidney injury and vancomycin/piperacillin/tazobactam in adult patients: A systematic review. *Internal and emergency medicine*. 2020;15:327-331

9. Chen XY, Xu RX, Zhou X, Liu Y, Hu CY, Xie XF. Acute kidney injury associated with concomitant vancomycin and piperacillin/tazobactam administration: A systematic review and meta-analysis. 2018;50:2019-2026

10. Blair M, Côté JM, Cotter A, Lynch B, Redahan L, Murray PT. Nephrotoxicity from vancomycin combined with piperacillin-tazobactam: A comprehensive review. *American journal of nephrology*. 2021;52:85-97

11. Bellos I, Karageorgiou V, Pergialiotis V, Perrea DN. Acute kidney injury following the concurrent administration of antipseudomonal β-lactams and vancomycin: A network meta-analysis. *Clinical microbiology and infection : the official publication of the European Society of Clinical Microbiology and Infectious Diseases*. 2020;26:696-705

12. Alshehri AM, Al Yami MS, Aldairem A, Alfehaid L, Almutairi AR, Almohammed OA, Badawoud AM. Evaluating the risk of acute kidney injury and mortality associated with concomitant use of vancomycin with piperacillin/tazobactam or meropenem in critically ill and non-critically ill patients: A systematic review and meta-analysis. *BMC infectious diseases*. 2025;25:36

13. Alaradi L, Albariqi N. Incidence of acute kidney injury (aki) in critically ill patients receiving concomitant vancomycin with piperacillin-tazobactam or cefepime; a systemic review and meta-analysis. 2025:8850666251323265

14. Yamashita Y, Kawaguchi H, Yano T, Sakurai N, Shibata W, Oshima K, Imai T, Yamada K, Nakamura Y, Nagayama K, Kakeya H. Risk factors for acute kidney injury in vancomycin and piperacillin/tazobactam combination therapy: A retrospective study. *Journal of infection and chemotherapy : official journal of the Japan Society of Chemotherapy*. 2021;27:1614-1620

15. Wuerger A, Bowden J, Mitchell A, Marler J. The effect of vancomycin and piperacillin-tazobactam on incidence of acute kidney injury in patients with obesity. *Antimicrobial agents and chemotherapy*. 2023;58:605-613

16. Venugopalan V, Maranchick N, Hanai D, Hernandez YJ, Joseph Y, Gore A, Desear K, Peloquin C, Neely M, Felton T, Shoulders B, Alshaer M. Association of piperacillin and vancomycin exposure on acute kidney injury during combination therapy. *JAC-antimicrobial resistance*. 2024;6:dlad157

17. Tomazini BM, Besen B, Taniguchi LU. Association between piperacillin/tazobactam use and acute kidney injury in critically ill patients: A retrospective multicentre cohort study. 2024;79:552-558

18. Su G, Xiao C, Cao Y, Gao P, Xie D, Cai Q, Nie S, Liu X, Lu F, Zhou Y, Hu Y, Li H, Yang Q, Wan Q, Liu B, Xu H, Li G, Weng J, Xu G, Chen C, Liu H, Shi Y, Zha Y, Kong Y, Tang Y, Shen J, Johnson DW, Xu X, Hou FF. Piperacillin/tazobactam and risk of acute kidney injury in adults hospitalized with infection without vancomycin: A multi-centre real-world data analysis. *International journal of antimicrobial agents*. 2023;61:106691
